# LIN7A is a major determinant of cell-polarity defects in breast carcinomas

**DOI:** 10.1186/s13058-016-0680-x

**Published:** 2016-02-17

**Authors:** Nadège Gruel, Laetitia Fuhrmann, Catalina Lodillinsky, Vanessa Benhamo, Odette Mariani, Aurélie Cédenot, Laurent Arnould, Gaëtan Macgrogan, Xavier Sastre-Garau, Philippe Chavrier, Olivier Delattre, Anne Vincent-Salomon

**Affiliations:** Institut Curie, PSL Research University, INSERM U830, 26 rue d’Ulm, 75248 Paris cédex 05, France; Département de Recherche Translationnelle, Institut Curie, PSL Research University, 26 rue d’Ulm, 75248 Paris cédex 05, France; Institut Curie, PSL Research University, CNRS UMR144, 26 rue d’Ulm, 75248 Paris cédex 05, France; Department of Pathology, Institut Curie, 26 rue d’Ulm, 75248 Paris cédex 05, France; Département de Pathologie and Centre de Ressources Biologiques Ferdinand Cabanne, Centre Georges François Leclerc, 1 rue Professeur Marion, BP 77980, 21079 Dijon cédex, France; Institut Bergonié, Service de Biopathologie, 229 cours de l’Argonne, 33076 Bordeaux, France

**Keywords:** Breast cancer, Micropapillary carcinomas, Cell polarity, LIN7A

## Abstract

**Background:**

Polarity defects are a hallmark of most carcinomas. Cells from invasive micropapillary carcinomas (IMPCs) of the breast are characterized by a striking cell polarity inversion and represent an interesting model for the analysis of polarity abnormalities.

**Methods:**

In-depth investigation of polarity proteins in 24 IMPCs and a gene expression profiling, comparing IMPC (n = 73) with invasive carcinomas of no special type (ICNST) (n = 51) have been performed.

**Results:**

IMPCs showed a profound disorganization of the investigated polarity proteins and revealed major abnormalities in their subcellular localization. Gene expression profiling experiments highlighted a number of deregulated genes in the IMPCs that have a role in apico-basal polarity, adhesion and migration. *LIN7A*, a Crumbs-complex polarity gene, was one of the most differentially over-expressed genes in the IMPCs. Upon LIN7A over-expression, we observed hyperproliferation, invasion and a complete absence of lumen formation, revealing strong polarity defects.

**Conclusion:**

This study therefore shows that LIN7A has a crucial role in the polarity abnormalities associated with breast carcinogenesis.

**Electronic supplementary material:**

The online version of this article (doi:10.1186/s13058-016-0680-x) contains supplementary material, which is available to authorized users.

## Background

Cell polarity is essential for normal mammary glandular epithelium organization. Whereas the absence of polarity is a hallmark of many breast cancers, invasive micropapillary carcinoma (IMPC) has a distinctive abnormal polarity, with an inverted apical pole [1]. Morphologically, IMPCs are characterized by clusters of cohesive carcinomatous cells in a micropapillary or tubular-alveolar arrangement, which appear to be suspended in clear spaces without any contact with the extracellular matrix (ECM). The mucin family protein MUC1, which is normally expressed at the apical membrane, is instead localized to the external surface of these carcinomatous cell clusters. This particular architecture has been attributed to a rotation of cell polarization and has been described as an inside-out growth pattern. Indeed, all IMPC cells have a well-defined MUC1^+^ apical pole that is covered by microvilli and oriented towards the stroma [1].

In addition to this specific pattern, IMPC tumors are associated with a striking propensity for lymphatic invasion and a high incidence of axillary lymph node metastasis, with reported rates more than 50 % higher than those of invasive carcinomas of no special type (ICNST) [2]. Despite this metastatic spread, the prognosis of IMPC is identical to that of ICNST [3].

In a previous genomic analysis of IMPC, we identified that several genes involved in polarity or ciliogenesis were mutated [4]. Given the specific pattern of IMPC and the identification of alterations in genes involved in polarity regulation, we thus hypothesized that IMPC could represent a good model for the analysis of polarity abnormalities in breast tumors.

There is evidence that polarity alterations have a central role in carcinogenesis [5]. However, the molecular mechanisms associated with the alteration of polarity proteins remain largely unknown. Indeed, only a small number of polarity genes have been shown to be involved in cancer development, including *Partitioning-defective 4* (*PAR4,* also known as *LKB1*), which is clearly recognized as a tumor suppressor gene, and *PAR3,* which is involved in enhancing metastases and breast carcinogenesis [6, 7]. Furthermore, the role of polarity genes in carcinogenesis is likely to be cell context-dependent, as described for isoforms of the gene encoding atypical protein kinase C (*aPKC*) [8].

IMPC morphological characterization is based on the inverted localization of MUC1; however, this protein is not involved in polarity maintenance and organization. We therefore performed an in-depth analysis of the expression of polarity proteins and transcripts in a series of IMPCs. We found that the gene encoding the polarity protein Lin-seven A (LIN7A) is specifically upregulated in IMPC and we therefore investigated the role of its over-expression in proliferation, invasion and the loss of apico-basal polarity using in vitro and in vivo experiments. Our results led us to demonstrate *LIN7A’*s role not only in IMPC but more generally in breast carcinogenesis.

## Methods

### Materials

We retrospectively selected 124 cases of invasive breast cancer - 73 pure IMPC and 51 ICNST (controls) - on the basis of the availability of paraffin blocks and frozen specimens. Cases came from the tumor banks of Institut Curie (46 IMPC and 51 ICNST), Centre Georges François Leclerc (20 IMPC) and Institut Bergonié (7 IMPC). The initial treatment was surgery in all selected cases. IMPC cases were confirmed on the basis of inside-out MUC1 staining at the inverted apical pole [1]. ICNST samples were selected as being estrogen receptor (ER)-matched and grade-matched with IMPC cases (Additional file 1: Table S1).

All experiments were performed in accordance with the French Bioethics Law 2004-800, the French National Institute of Cancer (INCa) Ethics Charter and after approval by the Institut Curie review board and the ethics committees of our institution (Comité de Pilotage du Groupe Sein). In that legal context, patients provided their informed consent for the use of their surgical tumor specimens for research. Data were analyzed anonymously.

### Cell culture

Non-tumorigenic MCF10A mammary epithelial cells (obtained from American Type Culture Collection; CRL-10317) were maintained as previously described [9]. To generate wild-type or LIN7A-expressing stable MCF10A cells, lentivirus infection was performed by transfecting HEK-293 T lentiviral packaging cells with empty vector or LIN7A-pCDH1-EF1 construct. At 48 h after transfection, retroviral supernatant was filtered through a 0.45-μm filter, supplemented with 5 μg/mL polybren and used to infect MCF10A cells (multiplicity of infection (MOI) = 3). MCF10A-vector and MCF10A-LIN7A cell lines were cultured in complete medium supplemented with 1 μg/mL of puromycin.

The human breast adenocarcinoma cell line MDA-MB-231 (HTB-26 obtained from American Type Culture Collection) cultured in DMEM culture medium containing 4 g/L glucose, 2 mM glutamine and 10 % FCS was stably transfected with LIN7A in a pCMV-3Tag-1A plasmid using Lipofectamine® LTX with Plus™ reagent (Invitrogen Carlsbad, CA), according to the manufacturers’ instructions (2.25 μg of DNA for 4 × 10^5^ cells) and cultured in the same medium supplemented with 0.6 mg/mL geneticin.

CAMA-1, an ER^+^ breast cancer cell line (HTB-21 obtained from American Type Culture Collection) was infected with empty vector or LIN7A-pCDH1-EF1 construct. CAMA-1-vector and CAMA-1-LIN7A cells were cultured in complete DMEM medium supplemented with 1 μg/mL of puromycin.

### 3D Matrigel™ cultures

Three-dimensional (3D) morphogenesis assays were performed with MCF10A stable cell lines. After trypsin treatment, 2 × 10^4^ cells/well resuspended in assay medium containing 5 ng/mL epidermal growth factor (EGF) [9] and 2 % Matrigel (Matrigel™ basement membrane matrix growth factor reduced, BD Biosciences, San Jose, CA, USA) were added to a Matrigel™-coated-6-well plate. Media were changed every 3 days for 11 days. Acini were fixed directly on alcohol-formaldehyde-acetic acid containing phloxine (AFA-phloxine fixative) at day 11 before paraffin embedding for immunostainings or lysed for immunoblotting.

### Invasion assay

Twenty-four-well Transwell BioCoat™ growth factor-reduced Matrigel™ invasion chambers (BD Biosciences, 8 μM pore size) were used for the invasion assay. After overnight serum starvation, cells were plated to the upper side of the Transwell device in serum-free medium, whereas the lower well contained regular 5 % horse serum (MCF10A) or 10 % FCS (MDA-MB-231, CAMA-1) culture medium to create a serum gradient. We seeded 5 × 10^4^ cells and stopped the experiment 48 h later. The remaining cells in the upper side of the Transwell device were removed and the invading cells at the bottom side of the Transwell device were fixed and counted.

### Antibodies and immunoblotting

After 11 days of 3D culture, Matrigel™ was dissolved and the whole-cell extracts were prepared in radioimmunoprecipitation assay (RIPA) buffer (50 mM Tris-HCl pH 7.4, 1 % NP40, 150 mM NaCl, 1 mM EDTA pH 8.0, 1 mM Na_3_VO_4_, 1 mM NaF) with protease and phosphatase inhibitor cocktail (Roche Bâle, Switzerland). Three-dimensional whole-cell extracts (30 μg) or 50 μg of tumoral proteins, extracted by 8 M urea, were resolved by 12 % SDS-PAGE and transferred onto nitrocellulose membrane (Bio-Rad, Marnes la Coquette, France).

Blots were incubated with anti-phospho-MEK1/2 (1:1 000 dilution, Cell Signaling, Boston, MA, USA), anti-MEK1/2 (1:2 000 dilution, Cell Signaling), anti-phospho-ERK1/2 (1:1 000 dilution, Cell Signaling), anti-ERK1/2 (1:1 000 dilution, Millipore, Darmstadt, Germany), anti-phospho-Akt (1:1 000 dilution, Cell Signaling), anti-Akt (clone 11E7, 1:1 000 dilution, Cell Signaling) or anti-LIN7A (1:1 000 dilution; Thermo Scientific, Rockford, IL, USA) antibodies. Beta-actin (1:10 000 dilution, Sigma Aldrich, Saint-Quentin Fallavier, France) was used as internal control for protein loading. Quantification of the expression was assessed by densitometry analysis.

### Immunohistochemistry

Formalin-fixed paraffin-embedded tissues sections were dried, deparaffinized and rehydrated according to standard procedures and stained with antibodies for 1 h. Staining was detected with the universal Vectastain Elite ABC peroxidase kit (Vector Laboratories, Burlingame, CA, USA), with diaminobenzidine (Dako A/S, Glostrup, Danemark) as chromogen. External controls were included for each antibody. Pictures were taken at G × 400 magnification.

Expression and cellular localization (subapical, apical, cytoplasmic, membranous, cell/cell: lateral membrane staining between two cells and basolateral) of polarity proteins was compared to that in normal cells. Antibodies used were directed against phospho-ezrin-radixin-moesin (p-ERM,1:200 dilution, pH6.1, BD Biosciences), GM130 (clone 35, 1:100 dilution, pH9, BD Biosciences), cell division control protein 42 (CDC42, 1:300 dilution, pH6.1, Lifespan Biosciences, Seattle, WA, USA), phospho-atypical PKCζ (p-aPKCζ, clone190D10, 1:250 dilution, pH6.1, Cell Signaling), protein associated with Lin seven 1 (PALS1, 1:50 dilution, pH9, Lifespan Biosciences), occludin (OCLN, 1:200 dilution, pH6.1, Lifespan Biosciences), Scribble (SCRIB, 1:50 dilution, pH6.1, Santa Cruz Biotechnology, Dallas, TX, USA), zonula occludens 1 (ZO-1, 1:500 dilution, pH6.1, BD Biosciences), β-catenin (clone 14/Beta-catenin, 1:200 dilution, pH6.1, BD Biosciences), E-cadherin (clone 4A2C7, 1:100 dilution, pH9, Invitrogen, Camarillo, CA, USA), KI-67 (clone MIB1, 1:100 dilution, pH6.1, Dako A/S) or active-caspase-3 (1:250 dilution, pH6.1, Cell Signaling).

### Gene expression analysis

The DNA microarrays used were the Human Genome U133 set (HG-U133 Plus 2.0, Affymetrix, Santa Clara, CA, USA), containing 54 613 probe sets. Experimental procedures for Gene Chip microarrays were performed according to the Affymetrix Gene Chip Expression Analysis Technical Manual using HG-U133 Plus 2.0 arrays. All microarray data were simultaneously normalized using the GC Robust Multi-array Average (GCRMA) package version 2.22.0 in the R 2.12.0 environment to assess the levels of expression of the gene probe sets. Genes were considered significantly deregulated between the two groups (Welch *t* test) when the *p* values were ≤0.05 after Bonferroni–Hochberg (BH) adjustment and the fold change was ≥1.5 (Additional file 2: Table S2). To identify the most relevant deregulated pathways, analysis was performed using the Database for Annotation, Visualization and Integrated Discovery (DAVID) and results were sorted by *p* value (*p* value with BH adjustment ≤0.05) (Additional file 3: Table S3). The most significant genes identified with the Welch *t* test were used to perform unsupervised clustering first of the training set, and subsequently of the validation set of tumors (standard Pearson correlation as similarity measure and centroid as linkage criteria). Receiving operating characteristic (ROC) curves were calculated to determine the specificity and sensitivity of the IMPC gene signature (Additional file 4: Figure S1) [GEO: GSE66418].

### Reverse transcription-quantitative PCR (RT-qPCR)

Total RNAs were extracted using the Trizol reagent, dosed and aliquoted by the Centre de Ressources Biologiques (CRB) from Institut Curie: 1 μg was reverse-transcribed with oligonucleotide-random hexamers using the High Capacity cDNA Reverse Transcription kit (Applied Biosystems, Life Technologies, Saint-Aubain, France). Assays-on-Demand for assessing the expression level of *LIN7A* and the control *TATA-binding protein* (*TBP*) genes were obtained from Applied Biosystems. qPCR was carried out in an ABI PRISM 7500 real-time thermal cycler using the Taqman master mix (Applied Biosystems). The relative mRNA expression was determined by the comparative cycle threshold method using the housekeeping gene *TBP. P* values were calculated using the Welch *t* test.

### Orthotopic mammary transplants

MDA-MB-231-LIN7A cells (3 × 10^6^ cells/mouse) were orthotopically injected into the mammary fat-pad of CB17-SCID female mice (7 weeks of age, Charles River Laboratories, L’Arbresle, France) and the tumor growth was followed for 15 weeks.

The care and use of animals were carried out according to European and National Regulations for the Protection of Vertebrate Animals used for Experimental and other Scientific Purposes (facility license number C75-05-18). They complies also with internationally established principles of replacement, reduction and refinement in accordance with the Guide for the Care and Use of Laboratory animals (NRC 2011) and Guidelines for the Welfare and Use of Animals in Cancer Research [10].

## Results

### IMPCs present abnormal localization of polarity proteins

**Fig. 1:**
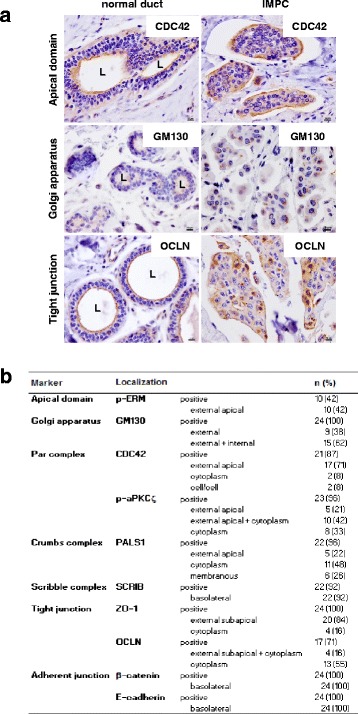
Polarity abnormalities in invasive micropapillary carcinoma (IMPC). **a** Representative views of cell-division control protein 42 (CDC42), cis-Golgi marker (GM130) and occludin (OCLN) immunostainings in three normal ducts (*left panel*) and in three IMPC (*right panel*). *Scale bars* 10 μm, *L* lumen. G × 400 magnification. **b** Analysis of polarity protein expression and subcellular localization in 24 IMPC. Expression and cellular localization (sub-apical, apical, cytoplasmic, membranous, cell/cell: lateral membrane staining between two cells and basolateral) of polarity proteins was compared to that in normal cells. *p-ERM* phospho-ezrin-radixin-moesin, *p-aPKCζ* phospho-atypical PKC, *PALS1* protein associated with Lin seven 1, *SCRIB* Scribble, *ZO-1* zonula occludens 1

We first compared 24 IMPCs with normal ducts and found that the expression and localization of several polarity proteins was abnormal (Fig. 1). Indeed, the apical domain markers were either absent, such as phospho-ERM, detected at the inverted apical pole, like CDC42 (Fig. 1a, upper panel) or observed in the cytoplasm (phospho-aPKCζ) (Fig. 1b). The protein associated with Lin-seven 1 (PALS1), which is part of the Crumbs complex that localizes to the apical membrane and is required for apico-basal cell polarity, was expressed in the cytoplasm in 48 % of the IMPCs. The Golgi matrix protein GM130 was delocalized from the juxta-nuclear area into the cytoplasm and produced a greater signal without any orientation in 62 % of the cases (Fig. 1a, middle panel). At the tight junctions, although ZO-1 was mainly expressed near the sub-apical inverted cell region (84 % of the cases), occludin (OCLN) was often mislocalized in the cytoplasm (55 % of the cases) (Fig. 1a, lower panel). The only proteins that were normally expressed in our screen were the adherens junction proteins E-cadherin and β-catenin, and the Scribble polarity complex protein SCRIB, which were localized at the basolateral domain, as expected.

Altogether, our results demonstrate a profound polarity disorganization in IMPC cells with complete inversion of the subcellular localization of some markers and major abnormalities for others.

### An IMPC-specific gene signature encompasses genes involved in the regulation of cell polarity, adhesion and migration

**Fig. 2:**
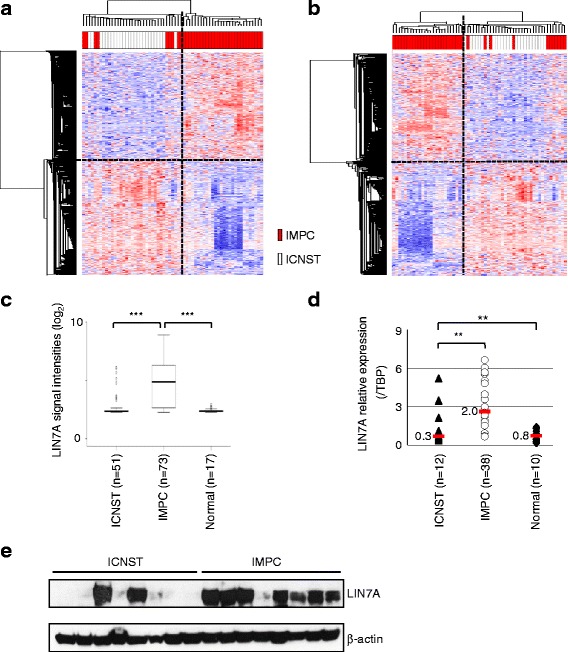
Specific invasive micropapillary carcinoma (*IMPC*) gene expression signature. **a**, **b** Human Genome U133 set (HG-U133 Plus 2.0) were simultaneously normalized using the GC Robust Multi-array Average package version 2.22.0 to assess the levels of expression of the gene probe sets. Unsupervised hierarchical clustering was performed with the differentially expressed 1108 genes in the training set (**a**) composed of 63 tumors (37 IMPC and 26 invasive carcinomas of no special type (*ICNST*)) and in the validation set (**b**) (61 tumors: 36 IMPC and 25 ICNST). Each *column* represents a different tumor and each *row* represents one of 1108 genes. *Red bars* IMPC, *white bars* ICNST. **c** *LIN7A* gene expression levels according to Affymetrix U133 Plus 2.0 signal in ICNST (n = 51), IMPC (n = 73) and normal breast tissues obtained from mammoplasties (n = 17). *P* values are based on the Welch two-sample *t* test; ***p value ≤0.001. **d** RT-qPCR on LIN7A expression in ICNST (n = 12, *black triangles*), IMPC (n = 38, *white circles*) and normal breast samples (n = 10, *black diamonds*). *LIN7A* gene expression levels are plotted on the *TBP* gene expression levels. Median values are indicated (*red bars*). *P* values were calculated with the Welch *t* test and are indicated *above the box-plot*; **p value ≤0.01. **e** Western-blot analysis of LIN7A expression in ICNST (n = 8) and IMPC (n = 8) tumors. Blots were incubated with anti-LIN7A or anti-β-actin antibodies as internal control for protein loading

To determine whether a specific gene signature could contribute to IMPC polarity abnormalities, we next performed gene profiling experiments with Affymetrix U133 plus 2.0 arrays to compare 37 IMPCs with 26 ICNSTs (Fig. 2). Using the Student *t* test (*p* value ≤0.05 and fold-change ≥1.5), the statistical analysis showed that the expression levels of 1108 genes was significantly different between the two groups of tumors (548 were downregulated and 560 were upregulated). The list of these genes (Additional file 2: Table S2) was used to perform unsupervised clustering in this series (the training set) (Fig. 2a). To investigate the robustness of this signature, we tested it in an independent series of cases (a further 36 IMPCs and 25 ICNSTs, corresponding to the validation set) (Fig. 2b). These analyses showed both high specificity and sensitivity in the training and in the validation sets (Additional file 4: Figure S1): area under the curve (AUC) = 0.88, specificity = 100 %, sensitivity = 76 % for the training set (left panel); AUC = 0.85, specificity = 100 %, sensitivity = 70 % for the validation set (right panel). We next performed an analysis of the IMPC gene signature using the DAVID functional annotation tool (Additional file 3: Table S3). The most represented gene ontology pathways specifically deregulated in IMPC were those involved in ECM constitution, cell-to-cell and cell-to-ECM adhesion, cytoskeleton organization, junction formation, migration and angiogenesis. Together, these genes play a crucial role in apico-basal polarity. Indeed, several genes involved directly in polarity complex formation, such as *LIN7A* and *SCRIB*, or in maintenance of tight and adherens junctions (*MPP7*, *CGN*, *CTNN* or *CLDN*) were upregulated. Conversely, other genes related to apico-basal polarity (*FOXO3, LOXL2, ZYX*), angiogenesis (*VEGFA*, *VEGFC*, *MMP2*, *LOX*), migration (*PLAU*, *TGFB1*, *SERPINB*5, *UNC5B*, *VIM*, *CAV2*, *CXCR4*) or coding for proteins involved either in cell-to-ECM adhesion (integrin, laminin, ankyrin repeat domain, actinin) or in ECM constitution (matrix metallopeptidase, serpin, collagen) were downregulated in IMPC. Genes related to actin cytoskeleton organization, such as *ACTA2*, *CDC42EP1*, *FLNA*, *SEPT02* or *PDLIM7* were also specifically deregulated in IMPC (Additional file 2: Table S2).

### The Crumbs-complex protein LIN7A is specifically over-expressed in IMPC

In this IMPC-specific gene signature, *LIN7A* was among the most differentially over-expressed genes and the first polarity-related gene, so it appeared to be a specific hallmark of this particular type of breast carcinoma. We confirmed the specific upregulation of LIN7A in IMPC (Fig. 2), compared to ICNST or normal breast tissue, at the levels of RNA (Fig. 2c and d) and protein (Fig. 2e).

### Over-expression of LIN7A disrupts MCF10A acini formation

To explore the role of LIN7A over-expression, we used the non-transformed mammary epithelial cell line MCF10A that is commonly used for in vitro polarity studies. After 11 days of 3D culture on reconstituted basement membrane (Matrigel™), these cells form completely polarized acini structures that recapitulate several aspects of glandular architecture in vivo [9].

**Fig. 3:**
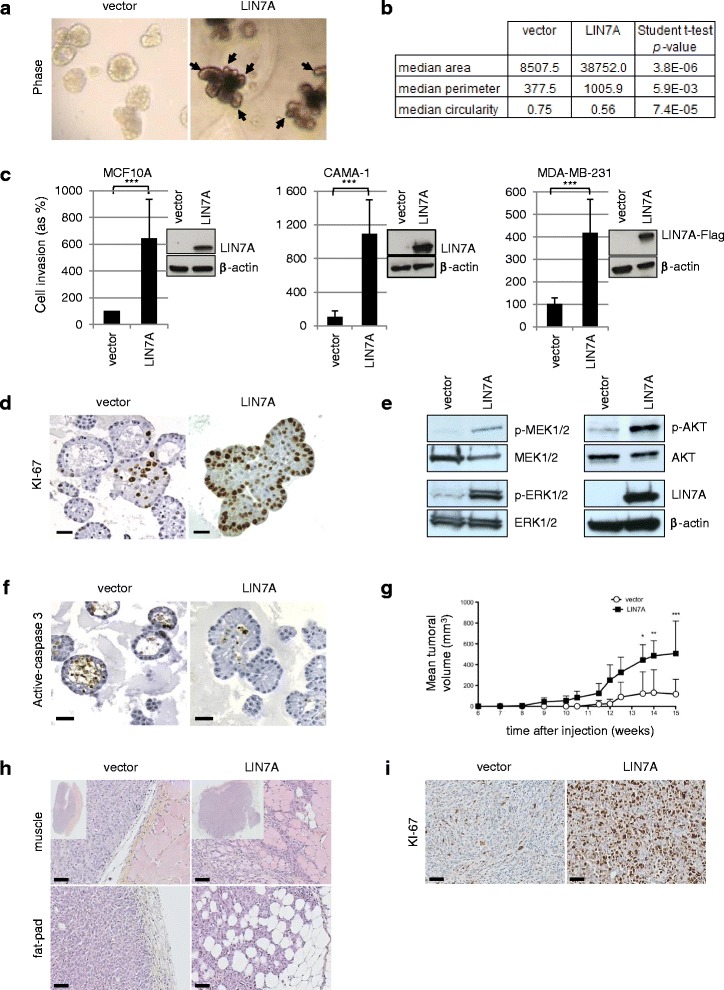
Over-expression of LIN7A disrupts apico-basal polarity, increases proliferation in vitro and enhancestumor growth in vivo. **a** Phase-contrast images of acinar structures obtained with MCF10A-vector (left) and MCF10A-LIN7A (right) cells (G × 630 magnification). Arrows point to the protrusions from the multi-acinar structures. **b** Area, perimeter and circularity of MCF10A-vector and MCF10A-LIN7A acini cultured in Matrigel™. Measurements were performed on five independent hematoxylin-and-eosin (HES) stained sections using Adobe Photoshop (Student *t* test). For circularity, a value of 1.0 indicates a perfect circle. **c** Quantification of LIN7A-expressing cells invasive potential (MCF10A, left panel; CAMA1, central panel; MDA-MB-231, right panel). ***p-value ≤0.001 (Student t test). **d** Representative views of KI-67 immunostaining in MCF10A-vector (left panel) and in MCF10A-LIN7A (right panel) acini. Scale bars 20 μm. G × 200 magnification. **e** Western-blot analysis on MCF10A-vector and MCF10A-LIN7A 3D whole-cell extracts. **f** Active-caspase-3 stainings of MCF10A-vector (left) and MCF10A-LIN7A (right) acini. Scale bars 20 μm. G × 200 magnification. **g** Tumor growth in CB17-SCID mice orthotopically injected into the mammary fat pad with MDA-MB-231-vector or MDA-MB-231-LIN7A cells. Curves represent the tumor volume according to time for MDA-MB-231-LIN7A (black squares) and the MDA-MB-231-vector control (white circles); *p value ≤0.05,**p value ≤0.01, ***p-value ≤0.001 (two-way analysis of variance, Bonferroni posthoc test). **h** Representative pictures of HES stained sections in mice muscle (top panel) or fat-pad (bottom panel) of the MDA-MB-231-vector (left panel) and MDA-MB-231-LIN7A (right panel) tumors, 15 weeks after injection. Scale bars 50 μm. **i** Representative pictures of KI-67 staining in MDA-MB-231-vector (left panel) and MDA-MB-231-LIN7A (right panel) tumors. Scale bars 50 μm

To analyze the consequences of LIN7A over-expression on polarity, MCF10A cells were either infected with the pCDH1-EF1-puro vector containing the *LIN7A* gene (MCF10A-LIN7A) or mock-infected (MCF10A-vector) before 3D culture (Fig. 3). At day 11, the MCF10A-vector cells exhibited completely polarized acini (Additional file 5: Figure S2a, left panel) with a regular single layer of cells organized around a well-formed hollow lumen, whereas the MCF10A-LIN7A cells disrupted normal 3D acinar morphogenesis by producing non polarized (Additional file 5: Figure S2a, right panel) larger and poly-lobulated masses with irregular borders that harbored large and numerous protrusions. These multi-acinar structures resemble those reported in mammary epithelial cells lacking-Par3 [7] and can similarly be interpreted as a massive invasion into the surrounding matrix (Fig. 3a and b). To confirm this hypothesis, in vitro invasion assays in Matrigel™-coated Transwell chambers were performed. Experiments have shown that MCF10A-LIN7A cells present a stronger invasive potential than the MCF10A-vector cells, as reflected by the higher number of invading cells in the LIN7A condition as compared to the control (Student *t* test, *p* value = 2.4 × 10^-4^) (Fig. 3c, left panel). The same results were obtained with the luminal ER^+^, cancerous but poorly invasive LIN7A-expressing CAMA-1 cell line (Student *t* test, *p* value = 1.7 × 10^-4^) (Fig. 3c, middle panel), suggesting that LIN7A expression was a promoter of increased invasive capacity in vitro.

The cell proliferation rate of these invasive multi-lobulated spherical masses as measured by KI-67 staining was strikingly high (45 % vs 10 % for MCF10A-vector, Fig. 3d). This was accompanied by increased levels of p-MEK/ERK1/2 and p-AKT proteins that are involved in cell-division pathways (Fig. 3e). These changes therefore indicate a pro-proliferative effect of LIN7A over-expression.

In addition, LIN7A over-expression disrupted acini formation (Fig. 3f). Indeed, in contrast to MCF10A-vector cells that formed typical acini in which lumen clearance was clearly present, significantly fewer acini with complete lumen were seen in the MCF10A-LIN7A multi-lobulated spherical masses (38 % vs 71 %, Student *t* test *p* value = 1.3 × 10^-9^) (Additional file 5: Figure S2b). Decreased lumen cell clearing was reflected in lower active caspase 3 staining (10 % vs 60 %) (Fig. 3f).

Altogether, these experiments show that the over-expression of LIN7A dramatically impairs epithelial cell glandular organization by inducing multi-dimensional invasive spherical masses, increasing proliferation and lumen filling.

### LIN7A promotes proliferation, tumor growth and invasion in vivo

To assess the LIN7A effect on invasion we searched for a cell line with low basal LIN7A expression, growing well in vitro, efficiently transfected and tumorigenic in mice. LIN7A is insufficient by itself to obtain in vivo tumors when over-expressed in the non-tumorigenic MCF10A cells (data not shown), so we over-expressed LIN7A in human breast adenocarcinoma MDA-MB-231 cells (MDA-MB-231-LIN7A). In vitro invasion assays have shown that MDA-MB-231-LIN7A cells presented a stronger invasive potential than the MDA-MB-231-vector cells, (Student *t* test *p* value = 6.3 × 10^-3^) (Fig. 3c, right panel). These results prompted us to perform in vivo experiments by injecting MDA-MB-231-LIN7A cells into the mouse mammary fat-pad.

Strikingly, MDA-MB-231-LIN7A tumors grew earlier and faster in the immunodeficient mice compared to those injected with mock-transfected cells (MDA-MB-231-vector) (Fig. 3g). MDA-MB-231-vector tumors were smaller than MDA-MB-231-LIN7A tumors (median: 4.9 mm^2^ vs 24.9 mm^2^; Student *t* test *p* value = 4.6 × 10^-2^) (Fig. 3h, upper panel). Histological examination showed that MDA-MB-231-vector mice tumors had well-limited borders (Fig. 3h, left panel) and central areas of necrosis in two out of four tumors (50 %). By contrast, MDA-MB-231-LIN7A tumors contained no necrosis in any of the 7 cases (0 %), had invasive borders with tumor cells invading as isolated cells or in solid sheets between adipocytes of the mice mammary fat-pads or muscles fibers (Fig. 3h, right panel) and were more proliferative, as reflected in a higher level of KI-67 staining (27.5 % vs 53.5 %, Student *t* test *p* value = 3.2 × 10^-2^) (Fig. 3i), suggesting that LIN7A plays a role in proliferation, which has previously never been described.

## Discussion

Apico-basal polarity alterations are increasingly being recognized as an important factor in tumor progression in breast cancers [11]. In this study, we have investigated the IMPC type of carcinoma, which has highly abnormal polarity, and we have defined a robust gene signature that encompasses the major polarity genes. Among the most deregulated genes in this signature, we identified the over-expression of *LIN7A* - which encodes a Crumbs-complex protein [12] - as being a specific hallmark of this particular type of breast carcinoma.

Crumbs, and other protein complexes including Par and Scribble, regulate apico-basal cell polarity by organizing compartments within the various cellular membranes. Their roles in mitosis, proliferation, cytoskeleton organization and in polarity maintenance are highly inter-related. Crumbs and Par are located at the apical pole, whereas Scribble is found at the apico-basal pole. The Crumbs complex encompasses CRUMBS (CRB), PALS1-associated tight junction (PATJ), PALS1, ERM and LIN7A proteins. LIN7A is a small scaffold protein containing an L27 domain, which stabilizes PALS1 [12] and mediates heterodimerization with several membrane-associated guanylate kinase (MAGUK) proteins, and a PDZ domain [13]. This PDZ domain binds to many proteins that are essential for cell polarity, cell adhesion and cell signaling.

Contrary to several studies that have shown a possible association between protein members of the polarity complexes and oncogenes or tumor suppressor genes in breast carcinogenesis, like the cooperation of *PAR6*, *PAR3* and *SCRIB* with the oncogenes *ERBB2* [14], *RAS* [6] or the tumor suppressor gene *PTEN* [15], respectively, in this study, we show that LIN7A alone induces polarity defects.

IMPC, characterized by a distinctive abnormal polarity, belongs to the luminal B carcinoma subtype, which is associated with high proliferation [4, 16] and invasive capacities (more than 60 % of vascular and axillary lymph node invasion at diagnosis) [17]. Our functional studies mirror these patterns; LIN7A over-expression: 1) impairs epithelial cell glandular organization by inducing non polarized multi-dimensional invasive spherical masses without any lumen, 2) increases cell proliferation through MEK/ERK and PI3K/AKT pathway activation, and 3) increases cell invasion, by inducing the formation of large and numerous protrusions into the surrounding matrix. Experiments showing disruption of the basement membrane of normal glands have not yet been monitored to make conclusions about the impact of LIN7A on invasion, but these 3D Matrigel™ experiments associated with in vitro invasion assays performed with different cell lines and invasive tumors in the mouse fat-pad provide the first convincing evidence on its role.

In the MCF10A model we observed that the expression of LIN7A has three major consequences: increased proliferation, disorganization of the cell polarity and alteration of the lumen formation. The MDA-MB-231 is not an appropriate model to appreciate the two last changes but we also observed larger MDA-MB-231-LIN7A tumors as compared to the MDA-MB-231-vector tumors, possibly due to increased proliferation in this system. These changes therefore indicate a pro-proliferative effect of LIN7A in two different cell systems. This is indeed a new finding about the role of LIN7A in proliferation. Whether this pro-proliferative role is a direct effect of LIN7A on the cell cycle or an indirect consequence of polarity modifications that cannot really be explored in the MDA-MB-231 system remain to be determined. In particular, polarity proteins have been shown to regulate the traffic and/or cell localization of signaling molecules that may in turn impact on cellular responses to proliferative signal [7, 18].

Previous studies have shown that via PDZ-domain interactions, LIN7A could both restrict HERs to specific subcellular compartments (basolateral localization) and actively participate in their signaling [19, 20], thereby increasing proliferation. Although we did not observe any phosphorylation of epidermal growth factor receptors (EGFRs) in our model, it is possible that LIN7A over-expression in IMPC increases proliferation and invasion in this particular tumor type through the activation of another tyrosine-kinase receptor. Preliminary experiments showed activation of INSULIN R or AXL in LIN7A-expressing cells, as compared to the control (Additional file 6: Table S4). INSULIN R by recruiting different substrate adaptors such as the IRS family of proteins can activate the PI3K/AKT pathway, promoting cell survival and proliferation. We have already shown that 3D-cultured LIN7A-expressing MCF10A cells increased cell proliferation through PI3K/AKT pathway activation (Fig. 3e). Interestingly, AXL has already been shown as a promoter of invasion and metastasis in breast experimental models [21]. We can therefore hypothesize that AXL receptor could be activated upon LIN7A upregulation and through that mechanism could contribute to promote non-coordinated migration, leading to epithelial structure disruption and to tissue infiltration by cancer cells.

The mechanism that induces LIN7A over-expression is unknown. Neither mutations nor genomic amplification have been identified so far [4], but other mechanisms such as epigenetic modifications still need to be explored.

## Conclusion

In conclusion, we have identified for the first time a role for LIN7A in breast carcinogenesis. We have also shown that LIN7A over-expression alone is sufficient to induce the absence of lumen, a traduction of an abnormal apical specification, and an increased proliferation and invasiveness of cancer cells. Together, these observations emphasize the importance of the interplay of polarity proteins and their potential role in breast carcinogenesis. Although we have shown that LIN7A is over-expressed in IMPCs, which constitute a sub-type of rare breast carcinomas, our in vitro experiments in the MCF10A, CAMA-1 and MDA-MB-231 cell lines indicate that the effect of LIN7A over-expression on proliferation, invasion and tumor growth is subtype-independent. A better understanding of the molecular pathways that underlie polarity establishment and maintenance will therefore facilitate the development of innovative therapeutic strategies that could be effective against many types of breast cancer.

## Additional files

Additional file 1: Table S1. Characteristics of patients and tumors. (PDF 53 kb) Additional file 2: Table S2. List of specific deregulated genes in invasive micropapillary carcinoma (IMPC) (Student t test, p-value ≤0.05, fold-change ≥1.5). (PDF 253 kb) Additional file 3: Table S3. Database for Annotation, Visualization and Integrated Discovery (DAVID) analysis performed on genes specifically deregulated in invasive micropapillary carcinoma (IMPC). (PDF 12 kb) Additional file 4: Figure S1. Evaluation of specificity and sensitivity by receiver operating characteristic (ROC) curves. ROC plot for training (*left panel*) and validation (*right panel*) sets. The area under the curve (AUC) is indicated (*bottom right*) for each plot. (PDF 12 kb) Additional file 5: Figure S2. Apico-basal polarity defects. **a** Representative views of GM130 immunostainings in MCF10A-vector (*left panel*) and in MCF10A-LIN7A (*right panel*) acini . *Arrows* indicate the Golgi apparatus position compared to the nucleus. **b** Quantification of lumen numbers after 11 days of 3D culture of MCF10A-vector and MCF10A-LIN7A cells, evaluated on all acini and spherical masses visible in the dishes; ***p* value ≤0.01. (PDF 39 kb) Additional file 6: Table S4. Quantification of receptor tyrosine kinase array in LIN7A-expressing MCF10A cells. (PDF 15 kb)

## Abbreviations

aPKC: atypical protein kinase C
AUC: area under the curves
BH: Bonferroni–Hochberg
CDC42: cell division control protein 42
DAVID: Database for Annotation, Visualization and Integrated Discovery
DMEM: Dulbecco’s modified Eagle’s medium
ECM: extracellular matrix
ER: estrogen receptor
ERM: ezrin-radixin-moesin
FCS: fetal calf serum
GCRMA: GC Robust Multi-array Average
GM130: golgi matrix protein
ICNST: invasive carcinomas of no special type
IMPC: invasive micropapillary carcinoma
LIN7A: lin-seven A
PALS1: protein associated with lin-seven 1
PAR4: partitioning-defective 4
ROC: receiver operating characteristic
SCRIB: scribble
TBP: TATA-binding protein
ZO-1: zonula occludens 1
