# Supplementary material for: LIN7A is a major determinant of cell-polarity defects in breast carcinomas
Source: Breast Cancer Res. 2016 Feb 17;18:23. doi: 10.1186/s13058-016-0680-x (PMC4756502; doi:10.1186/s13058-016-0680-x)
Supplement: Additional file 1: Table S1. — Characteristics of patients and tumors. (PDF 53 kb) [file 13058_2016_680_MOESM1_ESM.pdf]

Supplementary Table 1: Patients and tumors characteristics

	Training set (n=63)			Validation set (n=61)		
	IMPC (n=37)	ICNST (n=26)	*p* -val	IMPC (n=36)	ICNST (n=25)	*p* -val
**follow-up (months)**						
med, intervalle	137 [18-505]	43 [1-124]		97 [13-320]	51 [4-155]	
**age at diagnosis (yrs)**						
med, intervalle	56 [36-84]	58 [27-85]		61 [34-84]	56 [38-78]	
< 50	12	7	*ns*	6	7	*ns*
≥ 50	25	19		30	18	
**ER**						
+	34	26	*ns*	32	25	*ns*
-	3	0		4	0	
**ERBB2**						
3^+	6	16	*ns*	8	14	*ns*
ND	2	1		0	0	
**N**						
+	22	20	*ns*	20	18	*ns*
-	14	4		14	5	
ND	1	2		2	2	
**LVI**						
+	29	18	*ns*	26	18	*ns*
-	8	8		10	7	
**Grade**						
1	3	3	*ns*	2	3	*ns*
2	15	8		18	8	
3	17	15		15	14	
ND	2	0		1	0	

ER: estrogen receptor - N: axillary lymph node invasion - LVI: lympho vascular invasion
ND: not done - *ns* : not significant - med: median - yrs: years
