# Supplementary material for: LIN7A is a major determinant of cell-polarity defects in breast carcinomas
Source: Breast Cancer Res. 2016 Feb 17;18:23. doi: 10.1186/s13058-016-0680-x (PMC4756502; doi:10.1186/s13058-016-0680-x)
Supplement: Additional file 2: Table S2. — List of specific deregulated genes in invasive micropapillary carcinoma (IMPC) (Student t test, p-value ≤0.05, fold-change ≥1.5). (PDF 253 kb) [file 13058_2016_680_MOESM2_ESM.pdf]

Supplementary Table 2: List of specific deregulated genes in IMPC

ID	mean IMPC	mean ICNST	*p*-val (BH)	FC	symbol gene
Genes up-regulated in IMPC					
100506492_at	8,0	4,2	4,0E-04	13,6	M41
10202_at	9,6	6,2	2,7E-03	10,7	DHRS2
AFFX-HUMRGE/M10098_5_at	10,2	7,3	4,1E-04	7,7	NA
283078_at	5,9	3,0	1,9E-03	7,6	MKX
79153_at	7,5	4,6	4,0E-05	7,4	GDPD3
55897_at	7,3	4,7	7,7E-05	6,2	MESP1
1733_at	6,7	4,2	1,5E-02	5,8	DIO1
253190_at	8,4	6,1	4,2E-03	4,9	SERHL2
10267_at	7,3	5,2	1,0E-04	4,3	RAMP1
7980_at	6,3	4,2	4,8E-02	4,3	TFPI2
8825_at	4,9	2,8	6,3E-05	4,3	LIN7A
AFFX-HUMRGE/M10098_3_at	8,8	6,7	5,3E-03	4,3	NA
AFFX-HUMRGE/M10098_M_at	9,8	7,7	2,0E-03	4,2	NA
2537_at	11,1	9,1	1,3E-03	4,2	IFI6
3292_at	5,5	3,5	8,1E-03	4,2	HSD17B1
221662_at	7,1	5,1	2,7E-02	4,1	RBM24
9518_at	8,1	6,0	4,2E-03	4,1	GDF15
267_at	7,6	5,6	8,9E-06	3,9	AMFR
290_at	8,5	6,6	1,4E-02	3,7	ANPEP
341640_at	4,9	3,0	3,3E-03	3,6	FREM2
3866_at	10,1	8,4	3,8E-02	3,4	KRT15
1522_at	7,7	5,9	3,1E-06	3,4	CTSZ
285489_at	5,3	3,6	1,1E-02	3,4	DOK7
57613_at	8,2	6,5	3,0E-03	3,4	KIAA1467
92747_at	5,9	4,3	3,9E-02	3,2	C20orf114
7869_at	6,7	5,0	1,7E-03	3,2	SEMA3B
51196_at	5,3	3,6	2,0E-03	3,2	PLCE1
4277_at	9,0	7,4	2,7E-04	3,1	MICB
84914_at	6,9	5,2	4,1E-04	3,1	ZNF587
60598_at	8,7	7,1	3,2E-03	3,0	KCNK15
346389_at	5,4	3,8	3,0E-03	3,0	MACC1
23314_at	5,6	4,1	1,5E-02	3,0	SATB2
3754_at	5,9	4,3	1,1E-02	3,0	KCNF1
10268_at	6,4	4,9	1,1E-02	2,9	RAMP3
3860_at	3,7	2,2	3,4E-02	2,9	KRT13
100506409_at	4,2	2,6	4,8E-04	2,9	LOC100506409
6019_at	5,3	3,8	1,2E-02	2,9	RLN2
AFFX-M27830_5_at	10,8	9,2	9,8E-03	2,9	NA
51473_at	5,6	4,1	2,8E-02	2,8	DCDC2
120224_at	6,3	4,8	1,1E-02	2,8	TMEM45B
8416_at	8,1	6,6	3,3E-03	2,7	ANXA9
5090_at	7,7	6,2	1,8E-04	2,7	PBX3
169981_at	4,8	3,4	6,3E-05	2,7	SPIN3
116362_at	6,6	5,2	3,9E-03	2,7	RBP7
400793_at	7,0	5,6	1,1E-02	2,7	C1orf226
130814_at	10,9	9,5	3,5E-05	2,6	PQLC3
4603_at	7,4	6,0	1,2E-03	2,6	MYBL1
286256_at	5,5	4,1	1,0E-02	2,6	LCN12
22996_at	10,5	9,2	2,1E-03	2,5	TTC39A
4889_at	3,9	2,6	2,7E-02	2,5	NPY5R
89822_at	3,6	2,3	3,4E-02	2,5	KCNK17
2731_at	4,0	2,7	4,3E-02	2,5	GLDC
9215_at	6,6	5,3	2,8E-04	2,5	LARGE
145873_at	3,8	2,5	3,0E-04	2,5	MESP2
1499_at	8,2	6,9	2,9E-03	2,5	CTNNB1
54852_at	4,2	2,9	2,2E-05	2,5	PAQR5
51337_at	6,6	5,3	1,0E-03	2,4	C8orf55
55711_at	5,3	4,0	9,0E-03	2,4	FAR2
79624_at	11,7	10,4	1,0E-03	2,4	C6orf211
135932_at	4,9	3,6	1,5E-02	2,4	TMEM139
387723_at	4,7	3,4	4,5E-04	2,4	LOC387723
163589_at	4,4	3,2	3,3E-03	2,4	TDRD5
79993_at	6,1	4,9	9,9E-03	2,4	ELOVL7
83990_at	5,2	3,9	9,9E-03	2,4	BRIP1
9365_at	4,5	3,3	1,3E-02	2,4	KL
100288092_at	3,4	2,1	8,2E-03	2,3	LOC100288092
94121_at	7,5	6,3	1,4E-02	2,3	SYTL4
54898_at	5,2	3,9	5,0E-02	2,3	ELOVL2
8800_at	5,7	4,5	1,7E-03	2,3	PEX11A
9906_at	4,6	3,4	1,1E-04	2,3	SLC35E2
2299_at	4,0	2,8	3,8E-02	2,3	FOXI1
5570_at	7,6	6,4	3,6E-02	2,3	PKIB
128218_at	6,9	5,7	2,5E-03	2,3	TMEM125
24147_at	6,5	5,3	1,2E-02	2,3	FJX1
9410_at	6,3	5,1	2,9E-03	2,3	SNRNP40
55005_at	9,3	8,1	8,6E-03	2,3	RMND1
56649_at	3,7	2,6	2,1E-02	2,3	TMPRSS4
202333_at	4,8	3,6	1,4E-04	2,3	CMYA5
100287482_at	5,0	3,8	2,7E-03	2,3	NA
145474_at	4,0	2,8	6,2E-04	2,3	LOC145474
4935_at	3,9	2,7	9,5E-03	2,3	GPR143
10923_at	11,5	11,0	3,0E-02	1,5	SUB1
9166_at	9,6	9,1	1,8E-02	1,5	EBAG9
3029_at	8,3	7,8	9,0E-03	1,5	HAGH
9550_at	6,7	6,2	1,8E-02	1,5	ATP6V1G1
8226_at	7,7	7,2	2,9E-02	1,5	HDHD1
334_at	9,8	9,3	9,0E-03	1,5	APLP2
Genes down-regulated in IMPC					
3226_at	4,8	7,1	9,0E-03	-4,7	HOXC10
4057_at	6,6	8,7	3,2E-02	-4,5	LTF
3872_at	5,2	7,3	3,3E-03	-4,3	KRT17
3861_at	5,8	7,8	3,3E-02	-3,9	KRT14
84189_at	4,3	6,1	2,2E-02	-3,7	SLITRK6
8483_at	8,1	9,9	8,8E-03	-3,5	CILP
10398_at	8,1	9,9	4,0E-05	-3,4	MYL9
1292_at	8,8	10,5	1,0E-04	-3,3	COL6A2
5315_at	9,6	11,3	2,3E-03	-3,3	PKM2
100124700_at	3,8	5,5	4,9E-03	-3,3	HOTAIR
727936_at	8,0	9,7	5,3E-03	-3,2	GXYLT2
7474_at	5,6	7,3	4,6E-03	-3,1	WNT5A
28451_at	3,7	5,4	3,5E-02	-3,1	IGHV3-9
4239_at	4,5	6,1	6,5E-03	-3,1	MFAP4
6273_at	3,0	4,6	7,8E-03	-3,1	S100A2
24141_at	4,1	5,7	1,7E-03	-3,0	C20orf103
1116_at	6,0	7,6	1,2E-02	-3,0	CHI3L1
163782_at	4,1	5,7	5,1E-03	-3,0	KANK4
7791_at	7,2	8,8	2,0E-04	-3,0	ZYX
72_at	5,6	7,2	5,3E-03	-3,0	ACTG2
2052_at	7,8	9,3	1,9E-02	-2,9	EPHX1
3852_at	3,0	4,5	1,0E-02	-2,9	KRT5
4314_at	5,5	7,0	7,7E-03	-2,8	MMP3
5268_at	2,8	4,2	1,4E-02	-2,8	SERPINB5
2316_at	9,0	10,5	6,2E-05	-2,8	FLNA
4313_at	9,5	11,0	4,4E-04	-2,8	MMP2
54829_at	9,7	11,2	3,5E-03	-2,7	ASPN
127435_at	5,9	7,4	2,2E-04	-2,7	PODN
3854_at	3,1	4,5	2,4E-02	-2,7	KRT6B
389084_at	5,5	6,9	2,8E-03	-2,7	C2orf82
5589_at	8,0	9,5	2,2E-03	-2,7	PRKCSH
56265_at	4,3	5,8	1,0E-02	-2,7	CPXM1
4924_at	6,7	8,1	9,3E-04	-2,7	NUCB1
4240_at	6,5	7,9	7,1E-06	-2,7	MFGE8
58485_at	7,0	8,4	1,8E-02	-2,7	TRAPPC1
972_at	8,2	9,6	1,5E-03	-2,6	CD74
7177_at	6,9	8,3	2,3E-02	-2,6	TPSAB1
122786_at	8,8	10,2	3,1E-06	-2,6	FRMD6
3561_at	4,8	6,1	1,2E-02	-2,6	IL2RG
4237_at	9,0	10,4	1,7E-03	-2,6	MFAP2
196051_at	5,5	6,9	4,9E-03	-2,6	PPAPDC1A
1289_at	9,3	10,7	3,0E-04	-2,6	COL5A1
23452_at	5,7	7,0	1,2E-04	-2,5	ANGPTL2
83483_at	6,7	8,0	3,2E-03	-2,5	PLVAP
84759_at	6,8	8,2	1,1E-02	-2,5	PCGF1
6876_at	10,2	11,5	1,6E-04	-2,5	TAGLN
AFFX-r2-Bs-thr-5_s_at	4,7	6,0	4,2E-03	-2,5	NA
4320_at	7,5	8,8	6,6E-03	-2,5	MMP11
5142_at	5,6	6,9	5,1E-03	-2,5	PDE4B
4046_at	5,2	6,5	2,7E-04	-2,5	LSP1
6423_at	10,9	12,2	8,2E-04	-2,5	SFRP2
2922_at	5,9	7,2	2,3E-02	-2,5	GRP
716_at	8,3	9,6	4,0E-04	-2,5	C1S
54587_at	8,5	9,8	2,7E-04	-2,4	MXRA8
1759_at	5,1	6,4	2,9E-03	-2,4	DNM1
1296_at	5,8	7,1	4,7E-03	-2,4	COL8A2
23708_at	4,3	5,6	1,0E-02	-2,4	GSPT2
4921_at	5,4	6,7	9,6E-03	-2,4	DDR2
2706_at	7,6	8,9	2,2E-02	-2,4	GJB2
100652898_at	11,2	12,5	4,7E-02	-2,4	NA
633_at	8,4	9,6	3,1E-06	-2,4	BGN
79838_at	6,4	7,7	3,1E-02	-2,4	TMC5
5327_at	8,1	9,4	1,7E-02	-2,4	PLAT
493869_at	7,0	8,3	1,6E-04	-2,4	GPX8
57333_at	6,0	7,2	1,0E-04	-2,4	RCN3
84940_at	2,9	4,2	2,5E-02	-2,4	CORO6
3678_at	7,1	8,4	1,8E-03	-2,4	ITGA5
28959_at	8,0	9,3	4,4E-03	-2,4	TMEM176B
2049_at	5,4	6,7	4,0E-05	-2,4	EPHB3
338773_at	4,6	5,8	6,0E-04	-2,4	TMEM119
1173_at	9,6	10,8	1,0E-03	-2,4	AP2M1
57717_at	4,3	5,5	1,5E-02	-2,4	PCDHB16
2534_at	5,5	6,7	2,8E-04	-2,3	FYN
8324_at	6,4	7,6	1,9E-03	-2,3	FZD7
1300_at	9,5	10,7	6,8E-03	-2,3	COL10A1

9636_at	11,5	10,3	4,0E-02	2,2	ISG15
92421_at	8,9	7,8	2,3E-03	2,2	CHMP4C
117177_at	6,3	5,2	4,8E-04	2,2	RAB3IP
55502_at	4,2	3,1	5,2E-03	2,2	HES6
374819_at	8,3	7,2	1,7E-02	2,2	LRRC37A3
7263_at	8,9	7,8	2,0E-04	2,2	TST
1326_at	7,3	6,2	7,0E-03	2,2	MAP3K8
51204_at	6,7	5,6	8,4E-03	2,1	TACO1
201164_at	5,6	4,5	6,3E-04	2,1	PLD6
26999_at	7,1	6,0	2,5E-03	2,1	CYFIP2
55286_at	4,3	3,2	4,2E-02	2,1	C4orf19
254887_at	5,4	4,3	8,6E-04	2,1	ZDHHC23
54033_at	5,4	4,3	1,9E-02	2,1	RBM11
2628_at	7,5	6,4	2,5E-02	2,1	GATM
29065_at	5,7	4,6	1,1E-03	2,1	ASAP1-IT
130576_at	5,7	4,7	3,8E-02	2,1	LYPD6B
50484_at	10,2	9,2	4,5E-04	2,1	RRM2B
94025_at	3,8	2,7	3,0E-02	2,1	MUC16
5770_at	7,7	6,6	1,9E-03	2,1	PTPN1
80221_at	7,8	6,7	1,2E-02	2,1	ACSF2
154141_at	8,7	7,6	1,1E-02	2,1	MBOAT1
5534_at	5,1	4,0	4,8E-04	2,1	PPP3R1
283212_at	4,5	3,4	3,4E-03	2,1	KLHL35
64089_at	6,3	5,2	2,3E-04	2,1	SNX16
51673_at	4,2	3,1	2,9E-02	2,1	TPPP3
100507399_at	5,0	4,0	4,9E-04	2,0	HCG8
84440_at	5,0	4,0	1,4E-04	2,0	RAB11FIP4
357_at	5,0	3,9	1,2E-02	2,0	SHROOM2
6398_at	6,1	5,1	1,0E-02	2,0	SECTM1
57530_at	7,0	5,9	5,4E-03	2,0	CGN
29946_at	7,3	6,3	6,3E-05	2,0	SERTAD3
29092_at	4,7	3,7	6,3E-03	2,0	HSPC157
776_at	6,1	5,1	4,4E-02	2,0	CACNA1D
84179_at	4,0	2,9	1,2E-02	2,0	MFSD7
7739_at	7,6	6,5	2,7E-02	2,0	ZNF185
81539_at	11,0	10,0	1,1E-03	2,0	SLC38A1
26049_at	5,5	4,4	1,3E-02	2,0	FAM169A
7022_at	6,7	5,7	9,7E-03	2,0	TFAP2C
645722_at	3,3	2,3	1,6E-02	2,0	LOC645722
84126_at	5,8	4,8	6,3E-05	2,0	ATRIP
6603_at	8,9	7,9	5,8E-03	2,0	SMARCD2
100287628_at	3,8	2,8	2,4E-02	2,0	LOC100287628
100288152_at	6,4	5,4	1,8E-02	2,0	LOC100288152
11011_at	6,0	5,0	4,0E-03	2,0	TLK2
54443_at	6,6	5,6	3,2E-02	2,0	ANLN
11059_at	11,1	10,1	3,4E-04	2,0	WWP1
22822_at	6,6	5,6	1,4E-02	2,0	PHLDA1
56704_at	4,5	3,5	2,0E-02	2,0	JPH1
3703_at	8,7	7,7	2,8E-04	2,0	STT3A
143098_at	5,6	4,7	5,2E-03	2,0	MPP7
8364_at	10,3	9,3	1,5E-02	2,0	HIST1H4C
27340_at	5,3	4,3	2,0E-03	1,9	UTP20
23171_at	10,2	9,2	4,5E-04	1,9	GPD1L
7764_at	10,8	9,8	1,3E-03	1,9	ZNF217
401264_at	5,6	4,7	7,7E-05	1,9	FLJ37798
57222_at	8,7	7,8	2,5E-03	1,9	ERGIC1
55653_at	6,5	5,5	1,1E-02	1,9	BCAS4
79611_at	5,0	4,1	2,4E-02	1,9	ACSS3
100507316_at	5,6	4,7	8,4E-03	1,9	LOC100507316
257000_at	3,6	2,7	4,7E-03	1,9	PLAC2
196394_at	5,6	4,7	4,7E-03	1,9	AMN1
1501_at	4,1	3,1	2,8E-02	1,9	CTNND2
100289341_at	3,6	2,7	3,3E-03	1,9	LOC100289341
1875_at	7,3	6,4	2,6E-03	1,9	E2F5
9050_at	7,3	6,4	1,7E-03	1,9	PSTPIP2
29028_at	8,0	7,0	8,6E-03	1,9	ATAD2
64426_at	8,3	7,4	3,8E-06	1,9	SUDS3
23530_at	7,2	6,3	7,5E-04	1,9	NNT
79022_at	10,0	9,0	1,8E-03	1,9	TMEM106C
157769_at	9,3	8,3	3,5E-05	1,9	FAM91A1
100128822_at	7,7	6,8	4,9E-04	1,9	LOC100128822
58489_at	7,8	6,9	9,5E-03	1,9	FAM108C1
8187_at	6,7	5,8	1,8E-02	1,9	ZNF239
25849_at	4,9	4,0	2,4E-02	1,9	PARM1
10560_at	10,2	9,3	9,0E-03	1,9	SLC19A2
23475_at	6,1	5,1	3,7E-02	1,9	QPRT
1062_at	4,7	3,8	3,2E-02	1,9	CENPE
151473_at	7,5	6,6	3,0E-02	1,9	SLC16A14
1345_at	14,5	13,5	1,1E-04	1,9	COX6C
81607_at	5,6	4,7	1,8E-02	1,9	PVRL4
51107_at	7,9	7,0	4,0E-05	1,9	APH1A
79669_at	6,3	5,4	2,7E-02	1,9	C3orf52
390205_at	4,2	3,3	4,4E-02	1,9	LRRC10B
56920_at	5,3	4,4	4,0E-02	1,9	SEMA3G
27090_at	6,3	5,4	5,4E-03	1,9	ST6GALNAC4
2746_at	9,2	8,3	1,7E-03	1,9	GLUD1
489_at	6,3	5,4	1,3E-02	1,9	ATP2A3
6320_at	2,9	4,1	9,5E-05	-2,3	CLEC11A
3671_at	7,3	8,5	7,0E-04	-2,3	ISLR
1805_at	5,1	6,3	1,1E-02	-2,3	DPT
6662_at	7,9	9,2	2,7E-02	-2,3	SOX9
3225_at	4,7	6,0	4,7E-02	-2,3	HOXC9
2006_at	3,8	5,0	2,8E-03	-2,3	ELN
7043_at	7,0	8,2	1,5E-04	-2,3	TGFB3
4035_at	5,8	7,0	4,0E-05	-2,3	LRP1
6525_at	5,3	6,5	7,7E-05	-2,3	SMTN
7094_at	6,0	7,2	4,5E-04	-2,3	TLN1
5360_at	7,2	8,4	3,1E-03	-2,3	PLTP
3212_at	7,5	8,7	3,9E-02	-2,3	HOXB2
80176_at	5,1	6,3	4,0E-05	-2,3	SPSB1
2200_at	7,1	8,3	6,0E-04	-2,3	FBN1
1462_at	10,4	11,6	8,5E-04	-2,3	VCAN
5118_at	9,2	10,3	3,7E-04	-2,2	PCOLCE
27018_at	10,5	11,7	2,4E-03	-2,2	NGFRAP1
10417_at	7,2	8,4	2,3E-04	-2,2	SPON2
4958_at	4,4	5,5	2,2E-02	-2,2	OMD
6695_at	7,0	8,1	8,1E-03	-2,2	SPOCK1
79953_at	4,0	5,1	3,0E-03	-2,2	TMEM90B
83468_at	7,3	8,4	3,5E-03	-2,2	GLT8D2
3726_at	7,6	8,8	2,1E-03	-2,2	JUNB
9509_at	4,8	6,0	9,7E-06	-2,2	ADAMTS2
4319_at	2,9	4,0	2,6E-02	-2,2	MMP10
3914_at	3,7	4,8	2,5E-02	-2,2	LAMB3
10234_at	6,8	8,0	3,9E-02	-2,2	LRRC17
84624_at	7,1	8,2	4,4E-03	-2,2	FNDC1
5021_at	4,0	5,1	6,3E-03	-2,2	OXTR
51339_at	5,4	6,5	1,3E-02	-2,2	DACT1
10948_at	6,9	8,0	3,9E-02	-2,2	STARD3
2014_at	6,9	8,0	1,5E-02	-2,2	EMP3
4811_at	6,7	7,8	1,6E-04	-2,2	NID1
4267_at	9,7	10,8	2,7E-04	-2,1	NA
4148_at	4,2	5,3	5,1E-03	-2,1	MATN3
83872_at	6,0	7,1	1,1E-02	-2,1	HMCN1
50509_at	4,3	5,4	4,0E-03	-2,1	COL5A3
399_at	5,1	6,2	2,3E-02	-2,1	RHOH
1291_at	5,7	6,8	1,1E-03	-2,1	COL6A1
80781_at	8,0	9,1	2,1E-05	-2,1	COL18A1
55365_at	6,9	8,0	1,8E-02	-2,1	TMEM176A
2331_at	7,5	8,5	4,6E-03	-2,1	FMOD
11117_at	5,6	6,7	7,2E-04	-2,1	EMILIN1
26585_at	7,1	8,1	2,5E-02	-2,1	GREM1
8815_at	9,6	10,6	1,4E-02	-2,1	BANF1
7070_at	7,8	8,9	7,6E-05	-2,1	THY1
165_at	10,4	11,5	3,2E-04	-2,1	AEBP1
1072_at	11,1	12,1	8,8E-03	-2,1	CFL1
151887_at	7,0	8,1	9,0E-03	-2,1	CCDC80
22801_at	3,4	4,5	6,0E-04	-2,1	ITGA11
4016_at	9,7	10,8	1,4E-04	-2,1	LOXL1
51186_at	9,3	10,3	4,0E-05	-2,1	WBP5
51148_at	6,9	8,0	3,7E-03	-2,1	CERCAM
10418_at	4,5	5,6	2,8E-03	-2,1	SPON1
1264_at	4,5	5,6	1,3E-02	-2,1	CNN1
5045_at	5,8	6,8	1,4E-04	-2,1	FURIN
8840_at	3,6	4,7	9,1E-04	-2,1	WISP1
2191_at	7,6	8,6	3,5E-03	-2,1	FAP
63923_at	3,5	4,6	2,9E-02	-2,1	TNN
2295_at	2,7	3,7	4,9E-04	-2,1	FOXF2
710_at	9,2	10,3	3,8E-04	-2,1	SERPING1
375295_at	5,7	6,7	2,1E-02	-2,1	LOC375295
80206_at	2,5	3,6	4,0E-03	-2,1	FHOD3
3694_at	3,2	4,2	2,6E-02	-2,1	ITGB6
5176_at	10,2	11,3	1,1E-03	-2,1	SERPINF1
7041_at	7,4	8,5	7,1E-04	-2,0	TGFB1I1
79652_at	7,0	8,0	1,4E-04	-2,0	TMEM204
28452_at	2,5	3,5	1,5E-02	-2,0	NA
23646_at	8,7	9,7	2,0E-03	-2,0	PLD3
10979_at	6,5	7,5	9,5E-05	-2,0	FERMT2
145438_at	5,2	6,2	4,7E-05	-2,0	C14orf82
84620_at	3,4	4,4	5,1E-03	-2,0	ST6GAL2
8775_at	8,1	9,1	1,1E-02	-2,0	NAPA
715_at	8,8	9,8	1,0E-03	-2,0	C1R
5780_at	4,8	5,8	4,9E-04	-2,0	PTPN9
59_at	11,5	12,5	6,9E-04	-2,0	ACTA2
94234_at	2,6	3,6	8,8E-03	-2,0	FOXQ1
178_at	9,5	10,5	2,2E-03	-2,0	AGL
23650_at	4,6	5,6	1,1E-02	-2,0	TRIM29
AFFX-r2-Bs-dap-5_at	7,9	8,9	1,5E-02	-2,0	NA
79192_at	4,7	5,7	2,8E-02	-2,0	IRX1
147495_at	5,9	6,9	1,3E-02	-2,0	APCDD1
9235_at	2,8	3,8	3,7E-03	-2,0	IL32
8038_at	5,6	6,6	9,6E-03	-2,0	ADAM12
100628315_at	5,3	6,3	1,1E-02	-2,0	NA
3339_at	5,6	6,6	3,5E-05	-2,0	HSPG2
3487_at	10,3	11,3	3,0E-02	-2,0	IGFBP4

730091_at	3,9	3,0	1,4E-03	1,9	LOC730091
140735_at	5,4	4,5	1,5E-02	1,9	DYNLL2
55039_at	8,1	7,2	2,5E-03	1,9	TRMT12
5357_at	7,2	6,3	4,4E-02	1,9	PLS1
283310_at	3,6	2,7	3,5E-02	1,9	OTOGL
54503_at	7,6	6,7	8,4E-03	1,9	ZDHHC13
11072_at	7,1	6,2	2,6E-02	1,9	DUSP14
5349_at	9,1	8,2	3,6E-03	1,9	FXYD3
116447_at	6,0	5,1	5,3E-03	1,9	TOP1MT
642475_at	6,9	6,0	6,7E-03	1,9	C8orf73
2830_at	3,0	2,1	1,7E-02	1,9	GPR6
863_at	4,3	3,4	1,8E-02	1,9	CBFA2T3
55274_at	7,0	6,1	4,4E-03	1,9	PHF10
84941_at	4,7	3,8	1,8E-02	1,8	HSH2D
92335_at	7,7	6,9	8,6E-04	1,8	STRADA
114569_at	12,6	11,7	5,2E-03	1,8	MAL2
219844_at	6,8	5,9	1,1E-03	1,8	HYLS1
23327_at	7,1	6,2	2,2E-02	1,8	NEDD4L
23594_at	7,7	6,8	2,6E-02	1,8	ORC6
440957_at	4,7	3,8	8,1E-04	1,8	C3orf78
79752_at	9,0	8,2	1,1E-03	1,8	ZFAND1
1999_at	8,3	7,4	1,5E-02	1,8	ELF3
56924_at	4,1	3,3	2,1E-02	1,8	PAK6
27250_at	10,1	9,2	1,5E-02	1,8	PDCD4
10513_at	8,7	7,8	1,7E-02	1,8	APPBP2
8694_at	7,9	7,0	2,8E-03	1,8	DGAT1
84795_at	7,0	6,2	6,9E-03	1,8	PYROXD2
55779_at	6,2	5,3	7,8E-03	1,8	WDR52
10023_at	5,0	4,1	2,1E-03	1,8	FRAT1
494513_at	3,8	3,0	1,4E-02	1,8	DFNB59
6867_at	8,5	7,7	7,8E-03	1,8	TACC1
123872_at	5,5	4,7	1,7E-02	1,8	LRRC50
10565_at	8,4	7,5	3,2E-03	1,8	ARFGEF1
374378_at	5,2	4,3	2,9E-02	1,8	GALNTL4
1174_at	5,4	4,5	5,2E-03	1,8	AP1S1
64768_at	5,4	4,6	7,6E-05	1,8	IPPK
64282_at	8,6	7,7	2,0E-03	1,8	PAPD5
128344_at	4,6	3,8	4,0E-02	1,8	C1orf88
100113407_at	4,8	3,9	8,2E-03	1,8	TMEM170B
58513_at	4,9	4,0	7,2E-04	1,8	EPS15L1
55250_at	9,5	8,6	3,4E-02	1,8	ELP2
26270_at	7,5	6,7	2,1E-03	1,8	FBXO6
6674_at	8,0	7,1	2,2E-02	1,8	SPAG1
51365_at	3,6	2,7	4,4E-02	1,8	PLA1A
928_at	13,1	12,3	2,8E-04	1,8	CD9
26504_at	5,8	5,0	8,2E-03	1,8	CNNM4
6820_at	5,1	4,2	2,0E-02	1,8	SULT2B1
25994_at	7,5	6,7	7,1E-03	1,8	HIGD1A
79818_at	9,7	8,8	2,8E-02	1,8	ZNF552
51368_at	7,1	6,3	7,7E-05	1,8	TEX264
55314_at	5,4	4,5	1,6E-02	1,8	TMEM144
79075_at	6,6	5,8	4,0E-02	1,8	DSCC1
7718_at	5,5	4,6	2,1E-02	1,8	ZNF165
64798_at	9,2	8,3	3,3E-02	1,8	DEPTOR
116028_at	6,6	5,8	2,5E-02	1,8	C16orf75
5291_at	5,4	4,6	1,7E-03	1,8	PIK3CB
55766_at	8,0	7,2	7,8E-03	1,8	H2AFJ
51110_at	8,3	7,4	1,7E-02	1,8	LACTB2
440335_at	3,8	3,0	6,1E-04	1,8	LOC440335
80314_at	3,2	2,4	2,3E-03	1,8	EPC1
4602_at	10,5	9,7	3,0E-02	1,8	MYB
63897_at	7,1	6,3	3,4E-02	1,8	HEATR6
9650_at	8,5	7,7	9,0E-03	1,8	MTFR1
137695_at	7,5	6,7	1,0E-02	1,8	TMEM68
201475_at	4,1	3,3	2,0E-02	1,8	RAB12
55181_at	7,6	6,8	2,2E-02	1,8	C17orf71
221078_at	6,9	6,1	5,7E-03	1,8	NSUN6
57822_at	3,0	2,2	5,5E-03	1,8	GRHL3
6827_at	8,4	7,6	8,6E-03	1,8	SUPT4H1
9543_at	4,2	3,4	4,3E-02	1,8	IGDCC3
54797_at	3,4	2,6	1,1E-03	1,8	MED18
57508_at	7,6	6,8	9,9E-03	1,8	INTS2
79885_at	5,9	5,1	1,0E-03	1,8	HDAC11
6697_at	8,3	7,5	3,0E-03	1,8	SPR
201266_at	9,1	8,3	3,6E-02	1,7	SLC39A11
54619_at	5,6	4,8	6,3E-05	1,7	CCNJ
57707_at	5,3	4,5	2,3E-03	1,7	KIAA1609
100652765_at	6,4	5,6	1,9E-03	1,7	NA
22874_at	5,9	5,1	4,0E-03	1,7	PLEKHA6
7064_at	7,2	6,4	4,0E-04	1,7	THOP1
2330_at	4,2	3,4	4,7E-02	1,7	FMO5
80705_at	3,5	2,7	5,3E-03	1,7	TSGA10
60370_at	5,4	4,6	1,0E-04	1,7	AVPI1
1366_at	9,3	8,5	6,0E-04	1,7	CLDN7
51421_at	9,1	8,3	2,3E-03	1,7	AMOTL2
1534_at	9,5	8,7	1,5E-02	1,7	CYB561
124790_at	5,0	4,2	6,3E-03	1,7	HEXIM2
10468_at	4,9	5,9	2,3E-02	-2,0	FST
2615_at	6,0	7,0	3,4E-03	-2,0	LRRC32
5654_at	10,0	11,0	2,0E-03	-2,0	HTRA1
AFFX-HSAC07/X00351_5_at	11,8	12,8	5,3E-03	-2,0	NA
8642_at	3,7	4,7	4,0E-05	-2,0	DCHS1
1397_at	9,0	10,0	2,5E-02	-2,0	CRIP2
283208_at	3,4	4,4	1,6E-04	-2,0	P4HA3
11135_at	4,6	5,6	1,4E-02	-2,0	CDC42EP1
4017_at	3,7	4,6	4,0E-04	-2,0	LOXL2
3908_at	5,4	6,3	1,3E-02	-1,9	LAMA2
2327_at	4,2	5,2	2,8E-02	-1,9	FMO2
56126_at	3,7	4,7	6,8E-03	-1,9	PCDHB10
148170_at	5,9	6,9	3,9E-02	-1,9	CDC42EP5
25925_at	6,1	7,0	8,8E-03	-1,9	ZNF521
7280_at	9,8	10,7	3,1E-03	-1,9	TUBB2A
27286_at	6,1	7,0	3,5E-03	-1,9	SRPX2
3486_at	7,9	8,9	4,6E-03	-1,9	IGFBP3
1809_at	6,1	7,0	6,0E-03	-1,9	DPYSL3
6598_at	6,9	7,9	2,2E-03	-1,9	SMARCB1
114899_at	6,9	7,8	1,4E-02	-1,9	C1QTNF3
22795_at	8,3	9,3	1,6E-03	-1,9	NID2
219654_at	7,5	8,4	3,0E-03	-1,9	ZCCHC24
2289_at	6,3	7,3	1,7E-02	-1,9	FKBP5
23266_at	5,5	6,4	9,2E-03	-1,9	LPHN2
84168_at	5,5	6,5	1,3E-03	-1,9	ANTXR1
1634_at	11,1	12,0	1,0E-02	-1,9	DCN
800_at	8,5	9,4	3,4E-04	-1,9	CALD1
25903_at	7,7	8,6	2,3E-03	-1,9	OLFML2B
AFFX-DapX-5_at	7,4	8,3	1,2E-02	-1,9	NA
2950_at	7,3	8,2	1,0E-02	-1,9	GSTP1
79971_at	5,4	6,4	2,5E-02	-1,9	WLS
26167_at	2,3	3,2	1,2E-02	-1,9	PCDHB5
9818_at	5,3	6,3	1,8E-03	-1,9	NUPL1
10516_at	6,3	7,2	1,0E-02	-1,9	FBLN5
6624_at	6,4	7,3	1,4E-04	-1,9	FSCN1
144165_at	6,2	7,2	1,1E-02	-1,9	PRICKLE1
83716_at	8,7	9,6	1,1E-02	-1,9	CRISPLD2
112464_at	6,3	7,2	3,2E-03	-1,9	PRKCDBP
1284_at	8,8	9,8	4,5E-04	-1,9	COL4A2
54867_at	7,6	8,6	1,5E-02	-1,9	TMEM214
4015_at	4,1	5,0	2,7E-03	-1,9	LOX
92291_at	5,4	6,4	3,5E-02	-1,9	CAPN13
10630_at	3,6	4,5	1,6E-04	-1,9	PDPN
26137_at	9,1	10,0	1,0E-03	-1,9	ZBTB20
1059_at	7,0	8,0	1,7E-03	-1,9	CENPB
1410_at	6,3	7,2	2,3E-02	-1,9	CRYAB
22904_at	4,6	5,5	3,2E-03	-1,9	SBNO2
11096_at	4,9	5,8	7,9E-03	-1,9	ADAMTS5
23090_at	6,2	7,1	4,0E-03	-1,9	ZNF423
25960_at	5,4	6,3	1,6E-03	-1,9	GPR124
7472_at	5,1	6,0	2,2E-02	-1,9	WNT2
7058_at	10,9	11,8	5,2E-03	-1,9	THBS2
8743_at	9,6	10,5	1,5E-02	-1,9	TNFSF10
91653_at	6,3	7,2	7,5E-03	-1,9	BOC
1290_at	11,0	11,9	5,6E-03	-1,9	COL5A2
5156_at	9,0	9,9	1,5E-02	-1,9	PDGFRA
53826_at	4,2	5,1	1,2E-02	-1,9	FXYD6
9945_at	5,1	6,0	2,1E-02	-1,9	GFPT2
3357_at	3,3	4,2	4,9E-03	-1,9	HTR2B
AFFX-ThrX-5_at	4,2	5,1	7,1E-03	-1,9	NA
404217_at	3,3	4,2	4,4E-03	-1,9	CTXN1
1627_at	6,4	7,3	4,1E-04	-1,9	DBN1
1436_at	7,1	8,0	1,0E-02	-1,9	CSF1R
2195_at	8,2	9,1	1,5E-02	-1,9	FAT1
253827_at	4,4	5,3	3,5E-03	-1,9	MSRB3
2260_at	6,5	7,4	9,1E-03	-1,9	FGFR1
1303_at	8,1	9,0	1,2E-02	-1,8	COL12A1
10472_at	4,4	5,3	4,5E-02	-1,8	ZNF238
9260_at	3,9	4,8	1,6E-05	-1,8	PDLIM7
1009_at	9,1	9,9	5,3E-03	-1,8	CDH11
1727_at	8,7	9,6	2,8E-04	-1,8	CYB5R3
55076_at	8,0	8,8	1,7E-02	-1,8	TMEM45A
220323_at	5,7	6,6	6,0E-04	-1,8	OAF
3223_at	9,9	10,7	1,1E-02	-1,8	HOXC6
3371_at	8,7	9,5	3,8E-02	-1,8	TNC
11082_at	3,9	4,8	3,6E-03	-1,8	ESM1
6038_at	7,4	8,3	9,3E-03	-1,8	RNASE4
7422_at	6,8	7,6	2,0E-03	-1,8	VEGFA
55568_at	6,4	7,3	5,7E-03	-1,8	GALNT10
64943_at	5,2	6,1	2,4E-02	-1,8	NT5DC2
3611_at	9,1	9,9	8,3E-04	-1,8	ILK
10262_at	7,0	7,8	1,2E-02	-1,8	SF3B4
55959_at	8,8	9,7	4,1E-02	-1,8	SULF2
7040_at	7,0	7,9	4,4E-02	-1,8	TGFB1
10409_at	8,9	9,8	1,1E-02	-1,8	BASP1
8424_at	2,8	3,7	4,0E-02	-1,8	BBOX1
57125_at	6,3	7,2	6,3E-05	-1,8	PLXDC1

254427_at	5,0	4,2	2,7E-02	1,7	C10orf47	9781_at	5,8	6,6	2,5E-03	-1,8	RNF144A
388588_at	5,9	5,1	1,1E-02	1,7	LOC388588	6591_at	8,1	8,9	6,0E-03	-1,8	SNAI2
83719_at	9,8	9,0	6,8E-04	1,7	YPEL3	871_at	8,3	9,2	4,6E-03	-1,8	SERPINH1
83451_at	6,5	5,7	4,0E-03	1,7	ABHD11	388_at	6,9	7,7	2,1E-02	-1,8	RHOB
51239_at	5,0	4,2	9,0E-03	1,7	ANKRD39	8406_at	7,7	8,6	2,9E-02	-1,8	SRPX
771_at	10,9	10,2	4,5E-02	1,7	CA12	8682_at	9,0	9,9	3,1E-06	-1,8	PEA15
10189_at	8,6	7,8	1,4E-02	1,7	THOC4	4131_at	7,0	7,8	1,3E-02	-1,8	MAP1B
118980_at	7,9	7,1	1,9E-02	1,7	SFXN2	10381_at	6,2	7,0	7,2E-03	-1,8	TUBB3
84933_at	9,0	8,2	9,0E-03	1,7	C8orf76	115098_at	6,5	7,4	4,0E-03	-1,8	CCDC124
2203_at	9,8	9,0	8,1E-03	1,7	FBP1	718_at	10,4	11,3	2,5E-02	-1,8	C3
84305_at	6,0	5,2	1,5E-03	1,7	WIBG	5328_at	7,4	8,3	3,8E-02	-1,8	PLAU
154791_at	6,8	6,0	1,1E-02	1,7	C7orf55	10370_at	8,2	9,0	1,9E-02	-1,8	CITED2
10247_at	8,3	7,5	1,9E-02	1,7	HRSP12	7317_at	10,6	11,4	1,0E-02	-1,8	UBA1
157567_at	8,4	7,6	6,2E-03	1,7	ANKRD46	56944_at	8,0	8,8	4,7E-04	-1,8	OLFML3
144233_at	5,7	4,9	4,4E-04	1,7	BCDIN3D	5396_at	7,7	8,6	1,6E-02	-1,8	PRRX1
100272228_at	3,6	2,8	9,9E-03	1,7	LOC100272228	26872_at	5,9	6,7	4,4E-02	-1,8	STEAP1
9520_at	9,0	8,2	1,4E-03	1,7	NPEPPS	50805_at	2,7	3,6	3,6E-02	-1,8	IRX4
55258_at	7,5	6,8	2,6E-02	1,7	THNSL2	2019_at	2,6	3,4	1,5E-02	-1,8	EN1
284611_at	9,0	8,2	3,3E-02	1,7	FAM102B	7754_at	3,3	4,1	1,6E-02	-1,8	ZNF204P
404550_at	4,0	3,3	2,5E-03	1,7	C16orf74	60_at	11,9	12,7	5,3E-03	-1,8	ACTB
90390_at	6,9	6,1	1,2E-02	1,7	MED30	2982_at	7,1	8,0	7,3E-03	-1,8	GUCY1A3
11056_at	4,8	4,1	4,5E-02	1,7	DDX52	9843_at	5,9	6,7	1,7E-02	-1,8	HEPH
54532_at	8,1	7,3	2,0E-03	1,7	USP53	6678_at	11,6	12,5	4,3E-04	-1,8	SPARC
441150_at	3,7	3,0	2,3E-03	1,7	C6orf226	1513_at	10,3	11,2	1,6E-02	-1,8	CTSK
285368_at	5,9	5,2	3,6E-02	1,7	PRRT3	6709_at	8,3	9,2	1,0E-02	-1,8	SPTAN1
163486_at	6,3	5,6	2,9E-02	1,7	DENND1B	6522_at	6,3	7,1	4,8E-02	-1,8	SLC4A2
79673_at	7,5	6,7	2,6E-02	1,7	ZNF329	1842_at	6,8	7,7	3,6E-02	-1,8	ECM2
56061_at	8,4	7,6	1,1E-04	1,7	UBFD1	94031_at	6,4	7,2	1,2E-02	-1,8	HTRA3
54344_at	8,9	8,1	1,7E-02	1,7	DPM3	23500_at	4,4	5,2	8,4E-03	-1,8	DAAM2
1984_at	8,9	8,1	4,1E-02	1,7	EIF5A	7077_at	9,9	10,7	3,6E-04	-1,8	TIMP2
51608_at	6,0	5,2	3,5E-04	1,7	GET4	6813_at	6,7	7,6	3,2E-02	-1,8	STXBP2
92610_at	7,2	6,5	2,3E-02	1,7	TIFA	123920_at	6,9	7,8	2,4E-03	-1,8	CMTM3
8493_at	5,8	5,0	4,8E-02	1,7	PPM1D	4638_at	9,2	10,1	5,9E-03	-1,8	MYLK
348235_at	9,0	8,2	4,8E-02	1,7	SKA2	131578_at	5,5	6,3	1,4E-02	-1,8	LRRC15
79170_at	8,2	7,4	2,5E-02	1,7	PRR15L	79654_at	5,7	6,6	1,1E-02	-1,8	HECTD3
11000_at	7,5	6,8	2,8E-02	1,7	SLC27A3	389336_at	2,9	3,7	1,7E-02	-1,8	C5orf46
4715_at	11,2	10,4	8,2E-03	1,7	NDUFB9	57537_at	3,7	4,5	3,1E-03	-1,8	SORCS2
100507345_at	6,0	5,3	1,3E-02	1,7	LOC100507345	64359_at	8,1	8,9	1,1E-02	-1,8	NXN
100128927_at	6,5	5,8	1,8E-02	1,7	ZBTB42	10752_at	3,2	4,0	3,7E-02	-1,8	CHL1
323_at	7,8	7,1	1,4E-02	1,7	APBB2	1277_at	13,0	13,8	6,1E-04	-1,8	COL1A1
124739_at	6,8	6,1	2,4E-02	1,7	USP43	400931_at	5,9	6,8	4,4E-02	-1,8	LOC400931
28957_at	9,5	8,7	1,9E-02	1,7	MRPS28	5919_at	8,9	9,7	2,0E-02	-1,8	RARRES2
50865_at	10,9	10,1	3,1E-02	1,7	HEBP1	409_at	4,9	5,7	2,1E-02	-1,8	ARRB2
10420_at	6,0	5,3	4,3E-02	1,7	TESK2	728264_at	4,3	5,1	1,5E-03	-1,8	LOC728264
51649_at	9,5	8,7	3,5E-02	1,7	MRPS23	5216_at	11,0	11,8	4,0E-03	-1,8	PFN1
26578_at	8,1	7,4	4,6E-03	1,7	OSTF1	5738_at	7,8	8,6	2,8E-04	-1,8	PTGFRN
163183_at	8,3	7,6	1,4E-02	1,7	C19orf46	116535_at	3,5	4,3	1,9E-02	-1,8	MRGPRF
100289274_at	3,6	2,9	9,6E-03	1,7	LOC100289274	83987_at	4,5	5,3	2,2E-03	-1,7	CCDC8
147923_at	6,0	5,3	8,3E-03	1,7	ZNF420	8434_at	6,9	7,7	2,0E-02	-1,7	RECK
22820_at	7,9	7,2	2,6E-03	1,7	COPG	57124_at	5,4	6,3	1,8E-02	-1,7	CD248
340277_at	6,9	6,2	1,1E-02	1,7	C7orf46	23037_at	5,1	5,9	2,7E-02	-1,7	PDZD2
147685_at	4,6	3,9	5,0E-02	1,7	C19orf18	79070_at	5,3	6,1	6,1E-04	-1,7	KDELC1
729013_at	6,5	5,7	1,8E-02	1,7	LOC729013	26227_at	4,1	4,9	4,7E-02	-1,7	PHGDH
533_at	10,1	9,4	1,7E-03	1,7	ATP6V0B	920_at	6,8	7,6	1,4E-02	-1,7	CD4
55163_at	8,0	7,2	1,6E-02	1,7	PNPO	27122_at	7,8	8,6	1,2E-02	-1,7	DKK3
401068_at	4,0	3,3	1,3E-02	1,7	LOC401068	23523_at	6,9	7,7	6,3E-05	-1,7	CABIN1
135154_at	5,0	4,3	3,5E-02	1,7	C6orf57	57685_at	5,5	6,3	4,2E-02	-1,7	CACHD1
26094_at	6,8	6,1	3,6E-03	1,6	DCAF4	30008_at	7,6	8,3	6,0E-03	-1,7	EFEMP2
54958_at	6,1	5,4	3,6E-02	1,6	TMEM160	301_at	9,8	10,6	4,5E-03	-1,7	ANXA1
57003_at	9,6	8,9	2,6E-03	1,6	CCDC47	51621_at	5,4	6,2	5,4E-04	-1,7	KLF13
987_at	9,5	8,8	1,6E-03	1,6	LRBA	9902_at	8,2	9,0	1,1E-03	-1,7	MRC2
28512_at	7,0	6,3	6,3E-03	1,6	NKIRAS1	23240_at	5,4	6,2	1,0E-02	-1,7	KIAA0922
84706_at	5,6	4,8	3,5E-02	1,6	GPT2	11167_at	10,7	11,5	5,0E-03	-1,7	FSTL1
5087_at	10,3	9,6	1,7E-02	1,6	PBX1	10123_at	7,0	7,8	4,7E-03	-1,7	ARL4C
137075_at	4,2	3,4	8,6E-03	1,6	CLDN23	2664_at	8,6	9,3	1,4E-02	-1,7	GDI1
51571_at	9,7	9,0	2,3E-03	1,6	FAM49B	58499_at	5,4	6,2	5,0E-02	-1,7	ZNF462
2582_at	7,3	6,6	3,1E-02	1,6	GALE	5358_at	10,1	10,9	1,7E-03	-1,7	PLS3
93164_at	3,7	3,0	6,2E-03	1,6	HTR7P1	2149_at	8,5	9,3	3,1E-03	-1,7	F2R
1536_at	6,1	5,4	4,4E-02	1,6	CYBB	3845_at	9,2	10,0	1,8E-02	-1,7	KRAS
286053_at	7,8	7,1	1,9E-02	1,6	NSMCE2	3945_at	10,9	11,6	1,1E-02	-1,7	LDHB
11068_at	7,5	6,8	2,8E-04	1,6	CYB561D2	6698_at	2,0	2,8	3,9E-02	-1,7	SPRR1A
388722_at	3,8	3,1	2,4E-02	1,6	C1orf53	81618_at	6,1	6,9	8,5E-03	-1,7	ITM2C
8759_at	5,1	4,4	1,9E-02	1,6	NA	634_at	3,3	4,1	4,3E-02	-1,7	CEACAM1
122970_at	7,7	7,0	3,5E-02	1,6	ACOT4	5518_at	8,4	9,1	1,3E-02	-1,7	PPP2R1A
374882_at	10,1	9,4	9,9E-03	1,6	TMEM205	10908_at	5,8	6,6	2,9E-02	-1,7	PNPLA6
54512_at	7,6	6,9	1,5E-02	1,6	EXOSC4	10174_at	4,7	5,5	3,7E-03	-1,7	SORBS3
8526_at	4,6	3,9	1,5E-02	1,6	DGKE	2309_at	8,7	9,4	8,6E-03	-1,7	FOXO3
55093_at	8,6	7,9	1,1E-02	1,6	WDYHV1	80790_at	6,7	7,4	1,9E-03	-1,7	CMIP
7266_at	8,9	8,2	1,3E-02	1,6	DNAJC7	337876_at	3,5	4,2	1,5E-02	-1,7	CHSY3
24138_at	7,8	7,1	1,5E-02	1,6	IFIT5	23213_at	9,3	10,0	2,1E-02	-1,7	SULF1
80237_at	7,1	6,4	3,1E-02	1,6	ELL3	5159_at	7,9	8,7	2,3E-03	-1,7	PDGFRB
23329_at	5,2	4,5	3,9E-02	1,6	TBC1D30	79586_at	5,7	6,4	1,7E-02	-1,7	CHPF
79176_at	5,2	4,5	3,2E-03	1,6	FBXL15	6811_at	5,6	6,3	1,3E-02	-1,7	STX5
55015_at	8,0	7,3	5,3E-03	1,6	PRPF39	4854_at	4,8	5,5	2,2E-04	-1,7	NOTCH3
284309_at	8,8	8,2	9,0E-03	1,6	ZNF776	57616_at	5,5	6,3	1,7E-02	-1,7	TSHZ3
84987_at	8,9	8,2	2,2E-02	1,6	C12orf62	4212_at	6,2	7,0	3,5E-02	-1,7	MEIS2
100506649_at	3,2	2,5	1,5E-02	1,6	LOC100506649	85358_at	6,0	6,7	6,6E-03	-1,7	SHANK3

51728_at	8,8	8,1	1,0E-02	1,6	POLR3K	57493_at	8,1	8,9	7,9E-04	-1,7	HEG1
28991_at	7,2	6,6	1,7E-02	1,6	COMMD5	81876_at	7,7	8,5	2,3E-02	-1,7	RAB1B
285382_at	3,8	3,1	3,9E-02	1,6	C3orf70	3912_at	9,1	9,9	8,1E-04	-1,7	LAMB1
123879_at	6,4	5,7	1,3E-02	1,6	DCUN1D3	9322_at	6,4	7,1	7,0E-03	-1,7	TRIP10
7696_at	5,6	4,9	3,3E-02	1,6	ZNF137P	285590_at	7,0	7,7	1,4E-03	-1,7	SH3PXD2B
55284_at	7,3	6,6	9,9E-03	1,6	UBE2W	4692_at	6,8	7,5	1,2E-02	-1,7	NDN
387923_at	3,0	2,4	2,9E-02	1,6	SERP2	283298_at	4,7	5,4	1,6E-02	-1,7	OLFML1
100533106_at	3,6	3,0	1,5E-02	1,6	NA	8407_at	8,5	9,2	9,5E-03	-1,7	TAGLN2
6584_at	7,2	6,6	1,6E-02	1,6	SLC22A5	10509_at	7,3	8,1	1,9E-03	-1,7	SEMA4B
11232_at	6,5	5,8	1,6E-02	1,6	POLG2	56849_at	3,2	4,0	3,2E-02	-1,7	TCEAL7
91419_at	6,7	6,0	1,8E-02	1,6	XRCC6BP1	9138_at	4,8	5,5	8,1E-03	-1,7	ARHGEF1
80853_at	5,8	5,1	4,3E-03	1,6	JHDM1D	64175_at	7,1	7,8	2,7E-04	-1,7	LEPRE1
23341_at	7,9	7,3	2,3E-04	1,6	DNAJC16	283316_at	3,9	4,6	4,5E-02	-1,7	CD163L1
100507376_at	5,0	4,4	1,5E-02	1,6	LOC100507376	5325_at	7,0	7,7	1,5E-02	-1,7	PLAGL1
4070_at	12,4	11,7	3,5E-03	1,6	TACSTD2	649_at	4,1	4,8	2,1E-04	-1,6	BMP1
7915_at	4,4	3,7	6,3E-05	1,6	ALDH5A1	10631_at	12,9	13,6	8,3E-03	-1,6	POSTN
126353_at	7,3	6,7	3,8E-02	1,6	C19orf21	7075_at	3,5	4,3	1,5E-02	-1,6	TIE1
55167_at	6,9	6,2	7,0E-03	1,6	MSL2	284119_at	7,0	7,7	7,1E-03	-1,6	PTRF
10390_at	8,4	7,8	5,6E-03	1,6	CEPT1	9076_at	4,0	4,7	3,5E-02	-1,6	CLDN1
11244_at	8,7	8,0	9,6E-03	1,6	ZHX1	117248_at	2,6	3,3	2,7E-02	-1,6	GALNTL2
23204_at	12,3	11,7	1,4E-04	1,6	ARL6IP1	221061_at	5,4	6,2	3,8E-02	-1,6	FAM171A1
51001_at	9,8	9,1	5,3E-03	1,6	MTERFD1	26022_at	6,4	7,1	3,7E-02	-1,6	TMEM98
23412_at	10,1	9,5	2,7E-02	1,6	COMMD3	1294_at	3,9	4,6	2,6E-03	-1,6	COL7A1
57460_at	6,6	5,9	2,1E-02	1,6	PPM1H	219699_at	8,6	9,3	1,1E-02	-1,6	UNC5B
10140_at	12,4	11,7	1,6E-02	1,6	TOB1	84327_at	5,5	6,2	7,7E-03	-1,6	ZBED3
904_at	4,5	3,8	6,0E-04	1,6	CCNT1	2151_at	3,9	4,6	1,2E-02	-1,6	F2RL2
5017_at	5,8	5,2	2,2E-04	1,6	OVOL1	6228_at	6,8	7,5	5,0E-02	-1,6	RPS23
7123_at	6,4	5,7	3,4E-02	1,6	CLEC3B	1472_at	5,6	6,3	9,6E-03	-1,6	CST4
9603_at	4,8	4,1	3,7E-02	1,6	NFE2L3	85456_at	4,6	5,3	4,9E-04	-1,6	TNKS1BP1
90673_at	4,4	3,7	9,9E-03	1,6	PPP1R3E	23648_at	6,4	7,1	6,4E-03	-1,6	SSBP3
3191_at	8,6	7,9	9,5E-05	1,6	HNRNPL	79033_at	5,0	5,7	3,7E-02	-1,6	ERI3
84501_at	3,4	2,8	1,7E-02	1,6	SPIRE2	57507_at	3,4	4,1	2,4E-02	-1,6	ZNF608
11236_at	10,4	9,8	3,7E-03	1,6	RNF139	25932_at	6,5	7,2	1,7E-02	-1,6	CLIC4
29984_at	7,0	6,3	2,1E-02	1,6	RHOD	90378_at	4,0	4,7	2,7E-02	-1,6	SAMD1
11277_at	6,8	6,1	6,2E-04	1,6	TREX1	8630_at	4,4	5,1	3,4E-02	-1,6	HSD17B6
58490_at	7,3	6,7	4,8E-03	1,6	RPRD1B	7130_at	6,9	7,6	3,3E-02	-1,6	TNFAIP6
6988_at	7,9	7,2	1,7E-03	1,6	TCTA	396_at	7,0	7,7	1,4E-02	-1,6	ARHGDIA
3304_at	10,4	9,7	1,6E-02	1,6	HSPA1B	3915_at	8,1	8,8	3,3E-03	-1,6	LAMC1
8895_at	10,5	9,9	7,3E-03	1,6	CPNE3	8613_at	8,1	8,8	1,3E-02	-1,6	PPAP2B
5496_at	8,3	7,7	1,3E-04	1,6	PPM1G	3399_at	8,3	9,0	1,1E-02	-1,6	ID3
91862_at	3,9	3,2	1,1E-02	1,6	MARVELD3	90187_at	5,1	5,8	7,1E-03	-1,6	EMILIN3
56655_at	6,8	6,2	3,3E-02	1,6	POLE4	25878_at	11,4	12,1	1,4E-02	-1,6	MXRA5
144577_at	5,0	4,3	1,5E-02	1,6	C12orf66	5831_at	7,5	8,1	9,0E-03	-1,6	PYCR1
10585_at	5,8	5,2	5,4E-03	1,6	POMT1	221749_at	7,4	8,1	4,2E-03	-1,6	C6orf145
10471_at	7,0	6,3	3,0E-04	1,6	PFDN6	54796_at	4,6	5,3	3,0E-02	-1,6	BNC2
286148_at	8,6	8,0	3,5E-03	1,6	DPY19L4	3661_at	7,6	8,3	5,4E-03	-1,6	IRF3
2766_at	3,4	2,7	3,7E-02	1,6	GMPR	84627_at	7,5	8,2	2,4E-02	-1,6	ZNF469
11162_at	5,7	5,0	4,8E-02	1,6	NUDT6	5524_at	7,8	8,5	7,3E-03	-1,6	PPP2R4
222223_at	4,8	4,1	2,0E-02	1,6	KIAA1324L	6464_at	7,8	8,5	1,9E-03	-1,6	SHC1
100507309_at	6,3	5,7	3,6E-02	1,6	LOC100507309	125950_at	4,7	5,4	2,9E-02	-1,6	RAVER1
158219_at	5,8	5,1	1,0E-02	1,6	TTC39B	3779_at	2,3	2,9	2,6E-02	-1,6	KCNMB1
57494_at	4,1	3,4	4,2E-03	1,6	RIMKLB	7424_at	5,5	6,2	1,2E-02	-1,6	VEGFC
8673_at	12,0	11,3	1,2E-02	1,6	VAMP8	2619_at	5,6	6,2	2,0E-02	-1,6	GAS1
55248_at	7,0	6,4	8,8E-03	1,6	TMEM206	682_at	8,5	9,2	4,8E-02	-1,6	BSG
55191_at	7,5	6,9	2,8E-03	1,6	NADSYN1	1639_at	7,6	8,3	2,9E-02	-1,6	DCTN1
51451_at	10,0	9,4	1,5E-02	1,6	LCMT1	6764_at	7,1	7,7	1,7E-03	-1,6	ST5
11112_at	7,7	7,0	4,0E-03	1,6	HIBADH	57658_at	6,5	7,2	3,6E-02	-1,6	CALCOCO1
1364_at	8,9	8,3	1,5E-02	1,6	CLDN4	2319_at	5,0	5,7	2,5E-02	-1,6	FLOT2
23761_at	7,9	7,3	4,3E-02	1,6	PISD	2687_at	2,8	3,5	3,3E-03	-1,6	GGT5
55319_at	6,1	5,4	2,8E-02	1,6	C4orf43	65997_at	5,1	5,8	5,3E-03	-1,6	RASL11B
5002_at	9,1	8,4	2,6E-02	1,6	SLC22A18	11100_at	8,4	9,0	3,8E-03	-1,6	HNRNPUL1
7391_at	2,9	2,2	2,2E-02	1,6	USF1	23770_at	4,7	5,4	4,9E-02	-1,6	FKBP8
4591_at	7,5	6,8	3,3E-02	1,6	TRIM37	114904_at	3,1	3,8	1,1E-02	-1,6	C1QTNF6
51422_at	5,2	4,5	3,8E-03	1,6	PRKAG2	401237_at	3,4	4,1	4,6E-03	-1,6	FLJ22536
125488_at	5,4	4,8	2,7E-02	1,6	TTC39C	23022_at	10,1	10,7	9,0E-03	-1,6	PALLD
2729_at	8,1	7,4	4,6E-03	1,5	GCLC	7171_at	6,2	6,8	1,9E-02	-1,6	TPM4
55793_at	7,9	7,3	2,9E-02	1,5	FAM63A	23371_at	7,1	7,8	1,5E-02	-1,6	TENC1
581_at	6,1	5,5	3,0E-03	1,5	BAX	4735_at	9,5	10,1	4,3E-03	-1,6	SEPT02
123263_at	5,2	4,6	7,2E-03	1,5	MTFMT	29882_at	6,0	6,7	4,6E-02	-1,6	ANAPC2
64417_at	8,3	7,6	8,5E-03	1,5	C5orf28	153642_at	3,5	4,2	5,0E-03	-1,6	ARSK
55571_at	8,5	7,9	8,9E-06	1,5	C2orf29	11259_at	6,5	7,2	9,9E-03	-1,6	FILIP1L
2530_at	7,7	7,1	1,8E-02	1,5	FUT8	1545_at	8,2	8,9	8,6E-03	-1,6	CYP1B1
286075_at	4,0	3,4	8,8E-03	1,5	ZNF707	8877_at	4,8	5,5	2,9E-02	-1,6	SPHK1
4913_at	6,9	6,3	1,7E-02	1,5	NTHL1	83442_at	9,2	9,9	8,3E-03	-1,6	SH3BGRL3
117145_at	7,4	6,8	2,3E-02	1,5	THEM4	3574_at	3,4	4,1	1,8E-02	-1,6	IL7
1147_at	7,4	6,7	6,9E-04	1,5	CHUK	6444_at	3,2	3,9	8,8E-04	-1,6	SGCD
54621_at	4,5	3,9	2,0E-04	1,5	VSIG10	90952_at	4,5	5,2	1,2E-02	-1,6	ESAM
27315_at	7,8	7,2	5,7E-04	1,5	PGAP2	2737_at	7,6	8,2	5,0E-02	-1,6	GLI3
10294_at	8,9	8,3	1,7E-02	1,5	DNAJA2	1944_at	5,1	5,8	2,7E-02	-1,6	EFNA3
23508_at	5,6	5,0	2,6E-02	1,5	TTC9	6734_at	7,4	8,0	2,5E-02	-1,6	SRPR
388507_at	3,5	2,9	9,5E-03	1,5	ZNF788	192668_at	3,8	4,4	3,6E-02	-1,6	CYS1
23368_at	6,2	5,6	8,0E-03	1,5	PPP1R13B	7431_at	12,9	13,5	1,3E-03	-1,6	VIM
100653022_at	3,1	2,5	2,9E-02	1,5	NA	10766_at	6,6	7,3	4,3E-02	-1,6	TOB2
399664_at	3,9	3,3	1,7E-02	1,5	MEX3D	114823_at	7,0	7,7	6,8E-03	-1,6	LENG8
57146_at	6,1	5,5	3,0E-02	1,5	TMEM159	811_at	7,2	7,8	4,9E-04	-1,6	CALR
9110_at	8,6	8,0	1,6E-02	1,5	MTMR4	7045_at	10,2	10,8	2,2E-02	-1,6	TGFBI
10538_at	6,7	6,1	2,8E-02	1,5	BATF	29995_at	7,9	8,5	2,5E-02	-1,6	LMCD1

23513_at	8,4	7,8	2,4E-02	1,5	SCRIB	57630_at	6,5	7,2	4,1E-03	-1,6	SH3RF1
60436_at	6,0	5,3	1,6E-02	1,5	TGIF2	7014_at	6,1	6,7	1,3E-02	-1,6	TERF2
117246_at	8,4	7,8	4,7E-02	1,5	FTSJ3	4643_at	4,2	4,9	1,6E-03	-1,6	MYO1E
84287_at	9,1	8,5	7,7E-03	1,5	ZDHHC16	79068_at	8,2	8,8	1,7E-02	-1,6	FTO
10434_at	11,8	11,2	1,7E-02	1,5	LYPLA1	1281_at	14,0	14,6	4,0E-03	-1,6	COL3A1
5768_at	7,5	6,9	1,0E-02	1,5	QSOX1	666_at	6,7	7,3	2,5E-02	-1,6	BOK
56993_at	6,3	5,7	3,1E-02	1,5	TOMM22	87_at	10,2	10,8	2,7E-03	-1,6	ACTN1
26958_at	5,1	4,5	4,3E-02	1,5	COPG2	79088_at	6,9	7,5	8,2E-03	-1,5	ZNF426
375341_at	4,0	3,3	2,1E-03	1,5	C3orf62	2335_at	11,9	12,6	6,4E-03	-1,5	FN1
119559_at	7,9	7,3	2,1E-03	1,5	SFXN4	4157_at	3,3	4,0	1,2E-02	-1,5	MC1R
55266_at	6,4	5,8	1,0E-02	1,5	TMEM19	25937_at	6,6	7,2	4,0E-03	-1,5	WWTR1
23252_at	4,5	3,9	6,3E-03	1,5	OTUD3	54453_at	10,3	10,9	1,6E-03	-1,5	RIN2
124936_at	6,9	6,3	4,5E-02	1,5	CYB5D2	152007_at	5,1	5,8	2,2E-02	-1,5	GLIPR2
57621_at	7,4	6,8	6,7E-03	1,5	ZBTB2	AFFX-ThrX-M_at	6,0	6,7	3,3E-02	-1,5	NA
100505761_at	6,2	5,6	5,0E-02	1,5	LOC100505761	5900_at	7,8	8,4	2,1E-03	-1,5	RALGDS
8738_at	5,9	5,3	9,8E-03	1,5	CRADD	9537_at	6,2	6,8	4,7E-02	-1,5	TP53I11
734_at	6,4	5,8	3,9E-02	1,5	OSGIN2	1307_at	8,8	9,4	2,9E-02	-1,5	COL16A1
4905_at	9,9	9,3	2,6E-03	1,5	NSF	54976_at	5,2	5,9	7,7E-04	-1,5	C20orf27
5889_at	6,9	6,3	2,4E-02	1,5	RAD51C	8086_at	5,7	6,3	3,6E-02	-1,5	AAAS
90956_at	7,4	6,8	2,0E-04	1,5	ADCK2	30846_at	5,9	6,5	8,6E-03	-1,5	EHD2
6877_at	5,0	4,4	2,1E-03	1,5	TAF5	2077_at	5,5	6,1	2,1E-02	-1,5	ERF
2317_at	10,3	9,7	3,0E-02	1,5	FLNB	79171_at	7,4	8,0	2,0E-02	-1,5	RBM42
84083_at	4,5	3,9	2,1E-02	1,5	ZRANB3	3910_at	5,3	5,9	3,0E-04	-1,5	LAMA4
83862_at	7,4	6,8	7,7E-03	1,5	TMEM120A	81_at	6,8	7,5	2,3E-02	-1,5	ACTN4
29841_at	7,5	6,9	2,2E-02	1,5	GRHL1	7447_at	3,0	3,6	3,4E-02	-1,5	VSNL1
79837_at	9,0	8,4	3,0E-03	1,5	PIP4K2C	4060_at	12,9	13,5	7,5E-03	-1,5	LUM
9969_at	9,5	8,9	2,6E-02	1,5	MED13	3137_at	8,8	9,4	3,1E-02	-1,5	HLA-J
9791_at	9,7	9,1	1,0E-02	1,5	PTDSS1	92292_at	2,4	3,1	3,3E-02	-1,5	GLYATL1
79622_at	9,3	8,7	3,7E-02	1,5	SNRNP25	55893_at	6,1	6,7	3,9E-02	-1,5	ZNF395
23366_at	7,0	6,4	2,3E-02	1,5	KIAA0895	7852_at	7,5	8,1	3,5E-02	-1,5	CXCR4
64769_at	6,3	5,7	9,0E-03	1,5	MEAF6	858_at	6,8	7,5	2,8E-02	-1,5	CAV2
24148_at	7,2	6,6	1,3E-02	1,5	PRPF6	4837_at	9,1	9,7	3,7E-02	-1,5	NNMT
140685_at	3,1	2,5	1,3E-02	1,5	ZBTB46	55245_at	5,6	6,2	2,2E-02	-1,5	UQCC
51460_at	4,6	4,0	1,9E-03	1,5	SFMBT1	3397_at	8,0	8,6	2,9E-02	-1,5	ID1
9562_at	8,0	7,4	3,3E-03	1,5	MINPP1	91614_at	3,4	4,0	1,5E-02	-1,5	DEPDC7
115_at	6,5	5,9	2,9E-02	1,5	ADCY9	1762_at	4,0	4,6	1,3E-03	-1,5	DMWD
8148_at	7,0	6,4	4,7E-03	1,5	TAF15	124930_at	4,3	4,9	2,7E-02	-1,5	ANKRD13B
9897_at	10,1	9,5	1,9E-02	1,5	KIAA0196	145864_at	4,6	5,2	8,5E-03	-1,5	HAPLN3
10099_at	8,3	7,7	9,9E-03	1,5	TSPAN3	27303_at	5,0	5,6	2,9E-02	-1,5	RBMS3
51141_at	8,7	8,1	1,6E-02	1,5	INSIG2	23743_at	3,9	4,5	9,0E-03	-1,5	BHMT2
219743_at	4,7	4,1	2,5E-03	1,5	TYSND1	4642_at	6,3	6,9	2,4E-03	-1,5	MYO1D
1040_at	8,7	8,1	1,0E-02	1,5	CDS1	2012_at	9,2	9,8	3,4E-02	-1,5	EMP1
29071_at	9,3	8,7	3,3E-03	1,5	C1GALT1C1	153830_at	6,4	7,0	2,1E-02	-1,5	RNF145
23269_at	6,8	6,2	5,4E-03	1,5	MGA	7025_at	3,7	4,3	2,7E-02	-1,5	NR2F1
100507291_at	2,9	2,3	3,4E-02	1,5	LOC100507291	25792_at	6,5	7,1	3,3E-02	-1,5	CIZ1
100506342_at	3,1	2,6	1,3E-02	1,5	LOC100506342	6455_at	7,8	8,4	1,7E-03	-1,5	SH3GL1
90007_at	4,7	4,1	2,4E-03	1,5	MIDN	6935_at	4,6	5,2	1,6E-03	-1,5	ZEB1
85415_at	8,7	8,1	4,0E-02	1,5	RHPN2	6196_at	5,5	6,1	1,2E-02	-1,5	RPS6KA2
2245_at	5,5	4,9	3,3E-02	1,5	FGD1	8522_at	3,9	4,5	7,1E-03	-1,5	GAS7
55851_at	8,8	8,2	2,8E-03	1,5	PSENEN	2983_at	4,5	5,1	1,9E-02	-1,5	GUCY1B3
55256_at	9,6	9,0	2,2E-02	1,5	ADI1	AFFX-r2-Bs-thr-M_s_at	6,5	7,1	2,0E-02	-1,5	NA
51004_at	5,7	5,1	1,5E-02	1,5	COQ6	10954_at	7,5	8,1	1,3E-02	-1,5	PDIA5
79789_at	6,4	5,8	4,4E-02	1,5	CLMN	6310_at	7,7	8,2	1,3E-02	-1,5	ATXN1
5753_at	4,8	4,2	3,6E-02	1,5	PTK6	4056_at	4,4	5,0	7,8E-03	-1,5	LTC4S
84284_at	7,7	7,1	2,6E-02	1,5	NTPCR	84617_at	8,9	9,4	2,1E-02	-1,5	TUBB6
51092_at	8,6	8,0	7,2E-03	1,5	SIDT2	11067_at	4,8	5,4	3,5E-02	-1,5	C10orf10
100506828_at	4,9	4,4	2,1E-02	1,5	LOC100506828	1265_at	7,9	8,5	7,5E-04	-1,5	CNN2
25870_at	7,8	7,2	3,0E-04	1,5	SUMF2	6830_at	6,2	6,8	4,4E-02	-1,5	SUPT6H
79139_at	10,3	9,8	7,0E-03	1,5	DERL1	79899_at	4,1	4,6	4,2E-03	-1,5	PRR5L
139065_at	3,6	3,0	4,7E-02	1,5	SLITRK4	57104_at	5,9	6,5	2,7E-02	-1,5	PNPLA2
100506686_at	5,3	4,7	2,7E-02	1,5	LOC100506686	2771_at	8,0	8,6	3,4E-02	-1,5	GNAI2
23423_at	10,7	10,1	2,9E-02	1,5	TMED3	10656_at	5,8	6,3	3,2E-02	-1,5	KHDRBS3
10313_at	11,1	10,5	4,6E-04	1,5	RTN3	23152_at	6,6	7,2	8,0E-05	-1,5	CIC
4683_at	9,2	8,6	2,5E-03	1,5	NBN	5899_at	7,5	8,1	4,7E-04	-1,5	RALB
10269_at	10,7	10,1	4,5E-04	1,5	ZMPSTE24	4053_at	7,9	8,5	1,0E-02	-1,5	LTBP2
114112_at	4,6	4,1	1,7E-02	1,5	TXNRD3	28987_at	6,3	6,9	2,2E-02	-1,5	NOB1
8943_at	6,7	6,1	9,3E-03	1,5	AP3D1	1808_at	10,0	10,6	1,2E-02	-1,5	DPYSL2
3416_at	7,2	6,6	3,7E-03	1,5	IDE	23645_at	6,0	6,6	1,2E-02	-1,5	PPP1R15A
4189_at	7,7	7,2	1,0E-02	1,5	DNAJB9	55384_at	2,5	3,1	3,0E-02	-1,5	MEG3
1020_at	6,7	6,2	3,3E-02	1,5	CDK5	9644_at	5,7	6,3	4,2E-03	-1,5	SH3PXD2A
84316_at	8,8	8,3	4,8E-02	1,5	LSMD1	6925_at	8,4	9,0	1,3E-02	-1,5	TCF4
573_at	7,3	6,8	3,4E-02	1,5	BAG1	8535_at	7,9	8,5	4,7E-02	-1,5	CBX4
5774_at	5,9	5,4	1,1E-02	1,5	PTPN3	1278_at	13,9	14,5	9,6E-03	-1,5	COL1A2
378708_at	7,1	6,5	4,7E-02	1,5	APITD1	2246_at	2,7	3,2	1,6E-03	-1,5	FGF1
126295_at	5,4	4,9	1,7E-02	1,5	ZNF57	9572_at	3,2	3,8	3,0E-02	-1,5	NR1D1
84866_at	5,2	4,7	3,9E-02	1,5	TMEM25	92249_at	5,5	6,0	3,1E-02	-1,5	LOC92249
114876_at	9,0	8,4	3,7E-02	1,5	OSBPL1A	3965_at	6,8	7,3	4,4E-02	-1,5	LGALS9
282997_at	3,3	2,7	4,0E-02	1,5	LOC282997	79832_at	7,3	7,9	8,6E-04	-1,5	QSER1
5471_at	6,9	6,3	5,4E-03	1,5	PPAT	8404_at	11,4	12,0	5,0E-02	-1,5	SPARCL1
55607_at	4,5	3,9	6,4E-03	1,5	PPP1R9A	8729_at	6,3	6,9	3,2E-03	-1,5	GBF1
23389_at	7,9	7,4	1,2E-02	1,5	MED13L	3398_at	9,4	10,0	4,7E-03	-1,5	ID2
3838_at	12,3	11,8	2,7E-02	1,5	KPNA2	4150_at	5,5	6,1	3,7E-02	-1,5	MAZ
55508_at	8,8	8,2	1,0E-02	1,5	SLC35E3	55679_at	3,7	4,3	2,0E-04	-1,5	LIMS2
55114_at	8,2	7,7	2,9E-03	1,5	ARHGAP17	9891_at	8,2	8,7	3,4E-02	-1,5	NUAK1
57805_at	6,2	5,6	3,3E-02	1,5	KIAA1967	7169_at	3,2	3,8	1,5E-03	-1,5	TPM2
54994_at	8,5	7,9	1,1E-02	1,5	C20orf11	1030_at	4,5	5,1	6,2E-03	-1,5	CDKN2B
8036_at	9,8	9,3	9,8E-03	1,5	SHOC2	57205_at	6,6	7,2	2,0E-02	-1,5	ATP10D

164091_at	5,4	4,8	2,1E-03	1,5	PAQR7	4188_at	3,3	3,9	9,0E-03	-1,5	MDFI
219347_at	3,3	2,8	2,5E-02	1,5	LOC219347	23396_at	6,4	7,0	1,9E-02	-1,5	PIP5K1C
6646_at	7,0	6,4	3,2E-02	1,5	SOAT1	116138_at	7,0	7,5	1,7E-02	-1,5	KLHDC3
9121_at	3,6	3,1	2,6E-02	1,5	SLC16A5	9968_at	6,1	6,7	2,0E-02	-1,5	MED12
126789_at	6,1	5,6	1,5E-02	1,5	PUSL1	2995_at	6,7	7,3	1,4E-02	-1,5	GYPC
830_at	9,5	8,9	3,9E-02	1,5	CAPZA2	1314_at	7,5	8,1	9,2E-03	-1,5	COPA
100289635_at	6,1	5,6	4,0E-02	1,5	ZNF605	2275_at	4,4	4,9	9,5E-05	-1,5	FHL3
51076_at	6,2	5,6	2,0E-03	1,5	CUTC	8563_at	6,0	6,6	3,5E-02	-1,5	THOC5
56906_at	6,1	5,5	3,9E-02	1,5	THAP10	91975_at	3,8	4,3	1,6E-02	-1,5	ZNF300
10238_at	7,7	7,1	4,4E-02	1,5	DCAF7	1306_at	9,7	10,2	2,5E-02	-1,5	COL15A1
AFFX-M27830_M_at	9,6	9,0	9,4E-03	1,5	NA	4674_at	2,9	3,5	2,6E-02	-1,5	NAP1L2
54977_at	7,7	7,2	9,3E-03	1,5	SLC25A38	25927_at	5,5	6,1	4,6E-02	-1,5	CNRIP1
65010_at	5,4	4,9	5,2E-03	1,5	SLC26A6	8507_at	7,0	7,6	1,7E-02	-1,5	ENC1
79674_at	3,4	2,8	2,5E-02	1,5	VEPH1	5228_at	4,2	4,7	1,1E-02	-1,5	PGF
11076_at	3,5	2,9	4,4E-02	1,5	TPPP	2824_at	5,0	5,6	4,2E-02	-1,5	GPM6B
55719_at	5,8	5,2	1,0E-04	1,5	FAM178A	8646_at	2,3	2,9	1,1E-02	-1,5	CHRD
28232_at	3,9	3,4	2,6E-02	1,5	SLCO3A1	283899_at	7,4	7,9	2,3E-02	-1,5	INO80E
79697_at	7,5	6,9	1,1E-04	1,5	C14orf169	58526_at	6,4	7,0	1,1E-02	-1,5	MID1IP1
9489_at	8,1	7,5	6,8E-03	1,5	PGS1	8321_at	4,9	5,4	1,3E-02	-1,5	FZD1
57674_at	7,1	6,5	3,0E-02	1,5	RNF213	441478_at	6,3	6,8	3,5E-02	-1,5	NRARP
400099_at	4,3	3,8	2,9E-02	1,5	LOC400099	953_at	5,2	5,7	1,3E-02	-1,5	ENTPD1
54869_at	7,2	6,6	3,8E-02	1,5	EPS8L1	639_at	5,5	6,0	1,9E-02	-1,5	PRDM1
84955_at	7,3	6,8	2,4E-02	1,5	NUDCD1	5549_at	4,1	4,6	2,6E-02	-1,5	PRELP
51606_at	9,2	8,7	1,7E-02	1,5	ATP6V1H	5754_at	7,1	7,6	2,4E-02	-1,5	PTK7
5158_at	3,1	2,6	1,2E-02	1,5	PDE6B	51450_at	4,4	4,9	3,0E-02	-1,5	PRRX2
11033_at	5,2	4,6	1,2E-02	1,5	ADAP1	9746_at	4,2	4,8	1,4E-02	-1,5	CLSTN3
118813_at	5,5	5,0	3,3E-03	1,5	ZFYVE27	10956_at	9,0	9,6	2,5E-02	-1,5	OS9
55074_at	8,7	8,2	1,4E-02	1,5	OXR1	6586_at	4,8	5,3	3,0E-02	-1,5	SLIT3
100131607_at	6,2	5,7	3,3E-02	1,5	LOC100131607	832_at	10,1	10,7	2,8E-04	-1,5	CAPZB
2960_at	8,6	8,1	7,3E-03	1,5	GTF2E1	23325_at	8,0	8,6	3,6E-03	-1,5	KIAA1033
8161_at	7,1	6,5	4,0E-02	1,5	COIL	8460_at	4,2	4,7	3,8E-02	-1,5	TPST1
338758_at	4,9	4,4	3,3E-02	1,5	LOC338758	1200_at	8,3	8,9	1,5E-02	-1,5	TPP1
528_at	8,4	7,9	2,1E-02	1,5	ATP6V1C1	1601_at	7,8	8,4	2,5E-02	-1,5	DAB2
85465_at	7,2	6,7	6,9E-03	1,5	EPT1	23385_at	8,6	9,1	2,3E-02	-1,5	NCSTN
153562_at	5,5	5,0	2,2E-02	1,5	MARVELD2	84993_at	6,7	7,2	1,3E-02	-1,5	UBL7
79132_at	5,2	4,7	2,7E-02	1,5	DHX58	7846_at	11,8	12,3	1,8E-02	-1,5	TUBA1A
6184_at	10,1	9,5	1,8E-03	1,5	RPN1	23683_at	5,7	6,3	1,5E-02	-1,5	PRKD3
80146_at	8,3	7,7	5,3E-03	1,5	UXS1	9935_at	10,0	10,6	1,2E-02	-1,5	MAFB

BH: Benjamini-Hochberg - FC: Fold change
