# Supplementary material for: LIN7A is a major determinant of cell-polarity defects in breast carcinomas
Source: Breast Cancer Res. 2016 Feb 17;18:23. doi: 10.1186/s13058-016-0680-x (PMC4756502; doi:10.1186/s13058-016-0680-x)
Supplement: Additional file 3: Table S3. — Database for Annotation, Visualization and Integrated Discovery (DAVID) analysis performed on genes specifically deregulated in invasive micropapillary carcinoma (IMPC). (PDF 12 kb) [file 13058_2016_680_MOESM3_ESM.pdf]

Supplementary Table 3: DAVID analysis performed on genes specifically deregulated in IMPC

**Annotation cluster**	**Nb of genes**	***p*-val (BH)**
Extracellular matrix	83	5.0E-26
Extracellular matrix part	39	1.7E-16
Cell adhesion	96	1.3E-12
Basement membrane	13	3.7E-05
Cell binding	8	2.5E-04
Actin cytoskeleton	36	6.7E-04
Endoplasmic reticulum	65	1.2E-03
Actin binding	11	1.2E-03
Repeat: LRR 12	19	1.3E-03
Cell motion and migration	53	1.8E-03
Blood vessel development and angiogenesis	33	3.1E-03
Repeat: LRR 11	19	4.4E-03
Adherens junction	23	6.3E-03
Vesicle lumen	11	1.3E-02
Anchoring junction	23	2.1E-02
Platelet alpha granule lumen	11	2.3E-02
Repeat: LRR 10	19	2.5E-02
Collagen metabolic process	9	2.8E-02
Tight junction	11	2.9E-02
Cytoplasmic membrane-bounded vesicle lumen	10	2.9E-02
Cell-cell junction	24	3.0E-02
Glycosaminoglycan binding	20	3.6E-02
Regulation of cell migration	23	4.1E-02
EGF-like region, conserved site	35	4.2E-02
Thyroglobulin type-1	7	4.7E-02

BH: Benjamini Hochberg
