# Supplementary figures and images for: LIN7A is a major determinant of cell-polarity defects in breast carcinomas
Source: Breast Cancer Res. 2016 Feb 17;18:23. doi: 10.1186/s13058-016-0680-x (PMC4756502; doi:10.1186/s13058-016-0680-x)

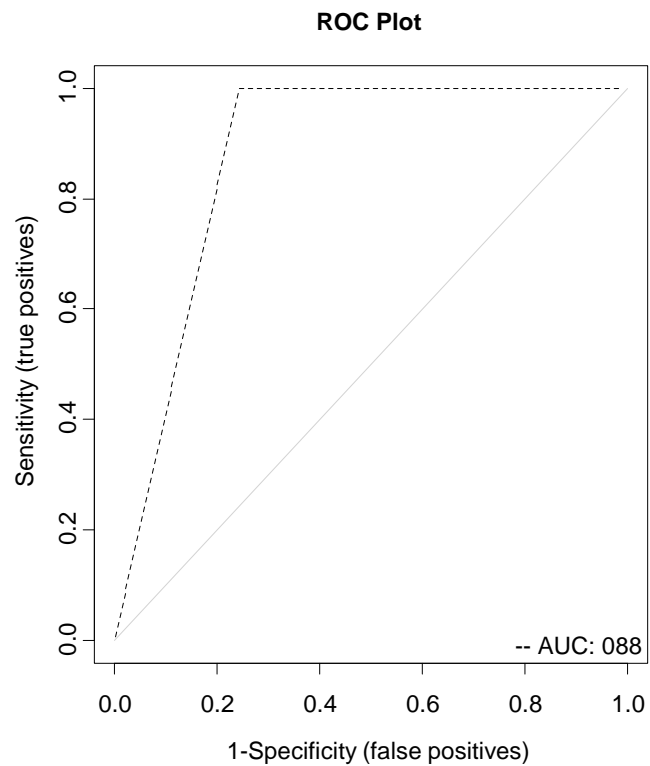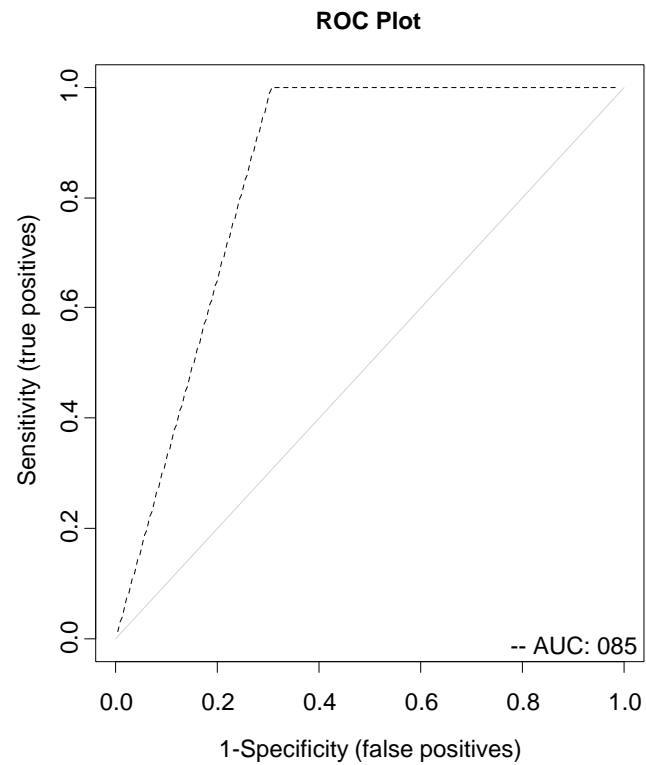

**Gruel *et al* Supplementary Figure 1**

Supplement: Additional file 4: Figure S1. — Evaluation of specificity and sensitivity by receiver operating characteristic (ROC) curves. ROC plot for training (left panel) and validation (right panel) sets. The area under the curve (AUC) is indicated (bottom right) for each plot. (PDF 12 kb) [file 13058_2016_680_MOESM4_ESM.pdf]

**a**

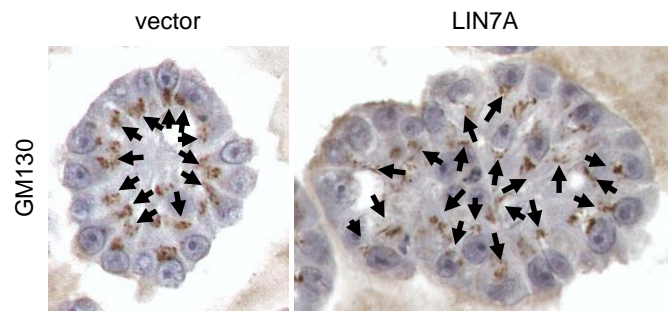

**b**

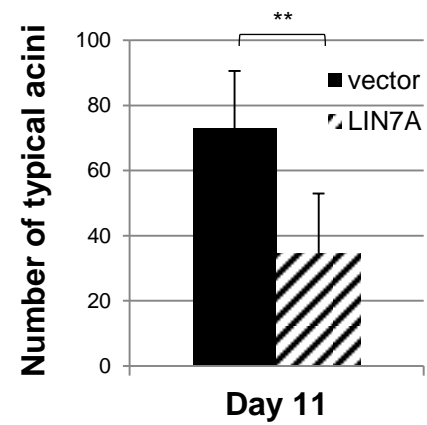

**Gruel *et al* Supplementary Figure 2**

Supplement: Additional file 5: Figure S2. — Apico-basal polarity defects. a Representative views of GM130 immunostainings in MCF10A-vector (left panel) and in MCF10A-LIN7A (right panel) acini . Arrows indicate the Golgi apparatus position compared to the nucleus. b Quantification of lumen numbers after 11 days of 3D culture of MCF10A-vector and MCF10A-LIN7A cells, evaluated on all acini and spherical masses visible in the dishes; **p value ≤0.01. (PDF 39 kb) [file 13058_2016_680_MOESM5_ESM.pdf]
