# Supplementary material for: LIN7A is a major determinant of cell-polarity defects in breast carcinomas
Source: Breast Cancer Res. 2016 Feb 17;18:23. doi: 10.1186/s13058-016-0680-x (PMC4756502; doi:10.1186/s13058-016-0680-x)
Supplement: Additional file 6: Table S4. — Quantification of receptor tyrosine kinase array in LIN7A-expressing MCF10A cells. (PDF 15 kb) [file 13058_2016_680_MOESM6_ESM.pdf]

Supplementary Table 4: Over-expression of LIN7A increases activation of receptor tyrosine kinases

	LIN7A / vector ratio	
	mean	SD
INSULIN R	2.72	0.22
AXL	1.76	0.35
IGF1R	1.08	0.45
ROR1	0.98	0.70
EGFR	0.88	0.12
EPHA2	0.77	1.20
EPHA3	0.74	0.03
ROR2	0.64	0.19
DTK	0.58	0.13
TIE1	0.57	0.19
EPHA4	0.57	0.03
EPHA5	0.42	0.80
CRET	0.38	0.08
MUSK	0.26	0.17
EPHB6	0.17	0.03
VEGFR2	0.16	0.00
MCSFR	0.15	0.11
VEGFR3	0.15	0.05
DDR1	0.13	0.02
ALK	0.10	0.03
VEGFR1	0.10	0.06
FGFR4	0.10	0.04
EPHB4	0.06	0.01
EPHB2	0.06	0.02
EPHA10	0.05	0.00
HGFR	0.05	0.02
RYK	0.05	0.03
TIE2	0.04	0.02
MER	0.03	0.02
EPHA1	0.03	0.00
ERBB2	0.03	0.01
TRKA	0.02	0.02
TRKB	0.02	0.00
MSPR	0.02	0.01
DDR2	0.01	0.01
TRKC	0.01	0.01
ERBB3	0.01	0.01
EPHA6	0.01	0.01
FGFR3	0.00	0.00
FLT3	0.00	0.00
FGFR2A	0.00	0.00
FGFR1	0.00	0.00
ERBB4	0.00	0.00
PDGFRA	0.00	0.00
PDGFRB	0.00	0.00
SCFR	0.00	0.00
EPHB1	-0.01	0.01
EPHB3	-0.03	0.00
EPHA7	-0.05	0.02

Human phospho-receptor tyrosine kinase arrays (R&D Systems, Minneapolis, MN) were incubated with 300 µg of whole protein extract obtained from MCF10A-LIN7A or MCF10A-vector cell lines. Arrays were quantified using ImageJ software.
